# Bmcc1s, a Novel Brain-Isoform of Bmcc1, Affects Cell Morphology by Regulating MAP6/STOP Functions

**DOI:** 10.1371/journal.pone.0035488

**Published:** 2012-04-16

**Authors:** Jessica Arama, Anne-Cécile Boulay, Christophe Bosc, Christian Delphin, Damarys Loew, Philippe Rostaing, Edwige Amigou, Pascal Ezan, Laure Wingertsmann, Laurent Guillaud, Annie Andrieux, Christian Giaume, Martine Cohen-Salmon

**Affiliations:** 1 Collège de France, Center for Interdisciplinary Research in Biology (CIRB)/Centre National de la Recherche Scientifique, Unité Mixte de Recherche 7241/Institut National de la Santé et de la Recherche Médicale U1050, Paris, France; 2 University Pierre et Marie Curie, ED, N°158, Paris, France; 3 MEMOLIFE Laboratory of Excellence and Paris Science Lettre Research University, Paris, France; 4 Equipe Physiopathologie du Cytosquelette, Institut National de la Santé et de la Recherche Médicale U836, Institut des Neurosciences, Université Joseph Fourier, Faculté de Médecine, Domaine de la Merci, La Tronche, France; 5 Institut Curie, Laboratory of Proteomic Mass Spectrometry, Paris, France; 6 Institut de Biologie de l'Ecole Normale Supérieure (IBENS), Institut National de la Santé et de la Recherche Médicale U1024, Paris, France; 7 Cell and Molecular Synaptic Function Unit, Okinawa Institute of Science and Technology, Okinawa, Japan; University of Montréal and Hôpital Maisonneuve-Rosemont, Canada

## Abstract

The BCH (BNIP2 and Cdc42GAP Homology) domain-containing protein Bmcc1/Prune2 is highly enriched in the brain and is involved in the regulation of cytoskeleton dynamics and cell survival. However, the molecular mechanisms accounting for these functions are poorly defined. Here, we have identified Bmcc1s, a novel isoform of Bmcc1 predominantly expressed in the mouse brain. In primary cultures of astrocytes and neurons, Bmcc1s localized on intermediate filaments and microtubules and interacted directly with MAP6/STOP, a microtubule-binding protein responsible for microtubule cold stability. Bmcc1s overexpression inhibited MAP6-induced microtubule cold stability by displacing MAP6 away from microtubules. It also resulted in the formation of membrane protrusions for which MAP6 was a necessary cofactor of Bmcc1s. This study identifies Bmcc1s as a new MAP6 interacting protein able to modulate MAP6-induced microtubule cold stability. Moreover, it illustrates a novel mechanism by which Bmcc1 regulates cell morphology.

## Introduction

The BCH (**B**NIP2 and ***C***dc42GAP ***H***omology)-domain-containing proteins have recently emerged as a new class of molecules involved in the regulation of cell dynamics through the engagement of specific Rho small GTPases. For instance, BNIP-2 induces cell protrusions by targeting Cdc42 [1]–[3] and promotes muscle differentiation [4]. p50RhoGAP modulates Rho and Cdc42 activity and controls cell morphology and cell migration [5]. BNIP-S targets RhoA and displaces p50RhoGAP, leading to RhoA activation, cell rounding and apoptosis [6], [7]. BP-GAP1 enhances RhoA activity, interacts with Cdc42 and Rac1, and controls cell morphology and migration [8], [9]. Recent works have also suggested that the functions of BCH-containing molecules could be more diverse. Indeed, BNIP-H (Caytaxin) interacts with the kidney-type glutaminase to regulate glutamate production and glutaminase trafficking [7], with the peptidyl-prolyl isomerase Pin1 to control neurite outgrowth [10] and with kinesin-1, an intracellular transport protein [11].

BMCC1 (**B**cl2, the adenovirus E1B 19 kDa interacting protein 2 and the Cdc42 GAP homology BCH **m**otif-**c**ontaining molecule at the **c**arboxy-terminal region **1**), also called PRUNE2, is a large molecule highly expressed in the brain as well as in spinal cord and dorsal root ganglia [12]–[14]. Overexpression of one of its isoforms, BNIP-XL (for BNIP-2 Extra Long), has been shown to promote the formation of short membrane protrusions, to inhibit RhoA and to suppress cell transformation initiated by Lbc, a RhoA-specific guanine nucleotide exchange factor [15]. Thus, a putative role for BMCC1 in the regulation of cytoskeleton dynamics as well as in apoptosis has been suggested. Interestingly, the BMCC1 transcript has been shown to be strongly upregulated in spontaneously regressing neuroblastomas [12], as well as in the neurodevelopmental Rett syndrome [16].

*BMCC1* encodes several isoforms whose expression pattern and subcellular localization are unknown. Here, we have identified Bmcc1s, a novel short isoform of Bmcc1 predominantly expressed in the mouse brain. We show that Bmcc1s localizes on intermediate filaments and microtubules in primary cultures of astrocytes and neurons, and interacts directly with MAP6 (aka STOP), a microtubule-associated protein responsible for microtubule cold stability [17], [18]. Moreover, we demonstrate that Bmcc1s overexpression inhibits microtubule stability through the displacement of MAP6 away from microtubules, resulting in the formation of membrane protrusions.

## Results

### Characterization of Bmcc1s, a novel brain Bmcc1 isoform

**Figure 1:**
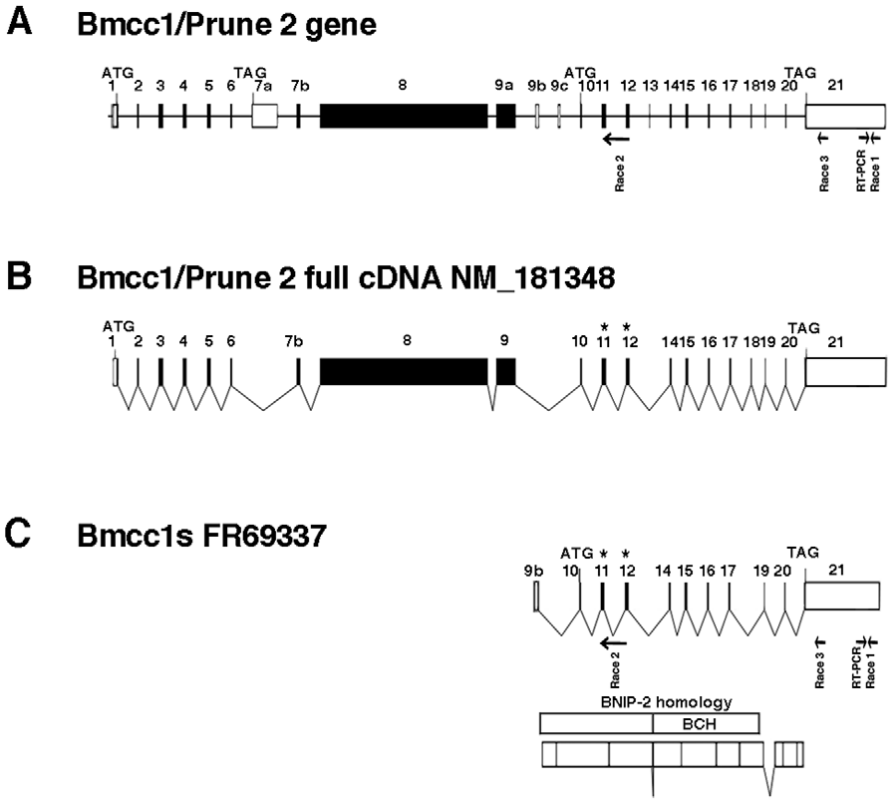
Structure of Bmcc1s. (A) Schematic representation of mouse Bmcc1 gene. Exons are boxed, in black for the coding sequence and in white for the 5′ and 3′ non-coding sequences. Primers for 5′ RACE and RTPCR experiments are indicated by arrows under exons 11, 12 and 21. (B) Schematic representation of mouse Bmcc1 transcript. (C) Schematic representation of Bmcc1s cDNA and protein. The BNIP2 homology and BCH domains are indicated. Asterisks show the antigenic peptides used to generate the Bmcc1s antiserum.

**Figure 2:**
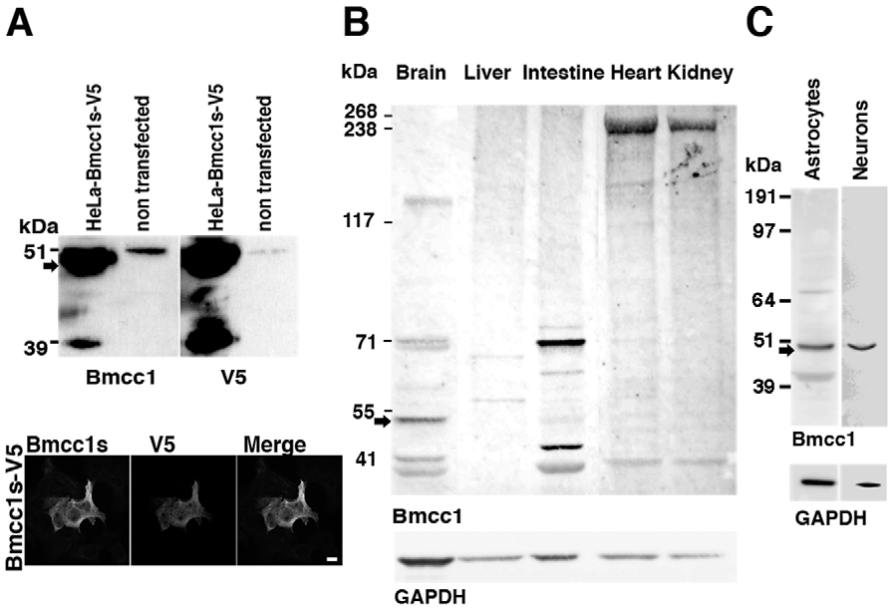
Immunodetection of Bmcc1s. (A) Immunoblot of Bmcc1s in lysates of HeLa cells transfected with a plasmid expressing Bmcc1s-V5. Similar profiles were obtained using the Bmcc1 antiserum or anti-V5 antibodies. Note that the Bmcc1 antiserum recognized an endogenous protein around 50 kDa (arrow) of the same size as Bmcc1s in untransfected HeLa cells. Immunostaining of HeLa cells transfected with a plasmid expressing Bmcc1s-V5, using either the Bmcc1 antiserum or anti-V5 antibodies. The antiserum detected only the V5 positive cells, and both signals overlapped. Scale bar: 100 µm (B) Immunoblot of endogenous Bmcc1 isoforms in mouse tissue lysates using Bmcc1 antiserum. GAPDH expression is shown as a loading reference. As in HeLa cells expressing Bmcc1s-V5, the Bmcc1 antiserum detected a band around 50 kDa (arrow) in the brain lysate that appeared specific to this tissue and was the most abundant among the Bmcc1 isoforms. (C) Immunoblot of endogenous Bmcc1 in primary cultures of astrocyte and neuron lysates at DIV7, using Bmcc1 antiserum. As found in brain tissues, a major band around 50 kDa was detected (arrow).

*BMCC1* encodes several isoforms [12], [13], [15]. A compilation of data from the literature and sequence databases is presented in figure 1, figure S1 (mouse gene) and figure S2 (human gene). Both human and mouse genes featured a comparable exon/intron structure and encoded multiple transcript isoforms, generated by alternative splicing and by the use of distinct promoters. In the mouse, *Bmcc1* cDNA variants encoded proteins containing either the N-terminal (N-ter) or the C-terminal (C-ter) end of the predicted full-length protein (up to 340 kDa) (Fig. S1). The sequence of the latter category corresponded mainly to the BNIP-2 homology domain, including the BCH domain (encoded by exons 14 to 17), which could vary depending on alternative splicing of exons 18, 19 or 20 (Fig. S1). Considering the numerous Bmcc1 isoforms, we aimed at defining those expressed in the mouse brain. 5′ RACE PCR performed on total adult mouse brain RNAs, starting from the 3′ end of Bmcc1 3′UTR (Material and methods and Fig. 1, Fig. S1A), led to the amplification of a unique 3.9 kbp product, which we named Bmcc1s for short Bmcc1 (EMBL accession number FR69337). Two additional 5′ Race PCR experiments starting from exon 12 and from the 5′ extremity of exon 21 did not extend the cDNA further (Material and methods and Fig. 1, Fig. S1A). Bmcc1s predicted open-reading frame mainly corresponded to the C-ter variant AK038997 (Fig. S1). It encoded a 323-amino acid predicted protein with a theoretical molecular mass of 37 kDa, which was 99% identical to AK038997 and contained a full BNIP-2 homology domain (Fig. 1). To evaluate the expression profile of Bmcc1s, we first performed RT-PCR experiments on the 3′ end of its 3′UTR (Fig. 1), using total RNA extracted from various mouse tissues (Fig. S3). Amplification occurred mainly in the brain, demonstrating that Bmcc1s expression is highly specific to this organ. To detect the cognate endogenously synthesized Bmcc1s protein, we raised a rabbit polyclonal antiserum directed against two peptides encoded by exon 11 and 12, which are present in all C-ter Bmcc1 isoforms (Material and methods and Fig. 1, Fig. S1). The specificity of this serum was tested by preincubating it with immobilized *in vitro* synthesized GST-Bmcc1s (Fig. S4, see Materials and Methods) and by immunostaining and immunobloting of HeLa cells transfected with a plasmid coding for Bmcc1s tagged with V5 (Material and Methods) (Fig. 2A). Both anti-V5 and anti-Bmcc1 antibodies revealed a band at the expected size of about 37 kDa, as well as a higher band around 50 kDa which could result from uncharacterized post-translational modifications of the protein (Fig. 2A). The 50 kDa band was also detected in untransfected HeLa cells, which endogenously express BMCC1 [12], [13]. Since one of the immunogenic peptides used to generate our antiserum was fully conserved in human (Fig. S2), this band may represent the endogenous BMCC1s protein. Finally, anti-Bmcc1 antibodies strongly detected the V5 positive HeLa cells, and both signals overlapped (Fig. 2A). Together, these results argued for the specificity of our Bmcc1 antiserum. The expression profile of Bmcc1s was next examined by Western blotting in various mouse tissue lysates (Fig. 2B), as well as in lysates of primary astrocyte and neuron cultures (Fig. 2C). In tissues, Bmcc1 antiserum recognized various patterns of bands. In particular, several bands were detected in brain extracts, demonstrating the expression of Bmcc1 isoforms longer than Bmcc1s. However, a 50 kDa band corresponding to the higher band detected in Bmcc1s-V5 expressing HeLa cells appeared as the most represented Bmcc1 variant and was not detected in the other tested tissues (Fig. 2B). The same major band was observed in the developing brain from birth to adult stage (Fig. S5) and in primary cultures of astrocytes and neurons (Fig. 2C). This Bmcc1 isoform should thus represent Bmcc1s.

### Bmcc1s localizes on microtubules and intermediate filaments in neurons and astrocytes

**Figure 3:**
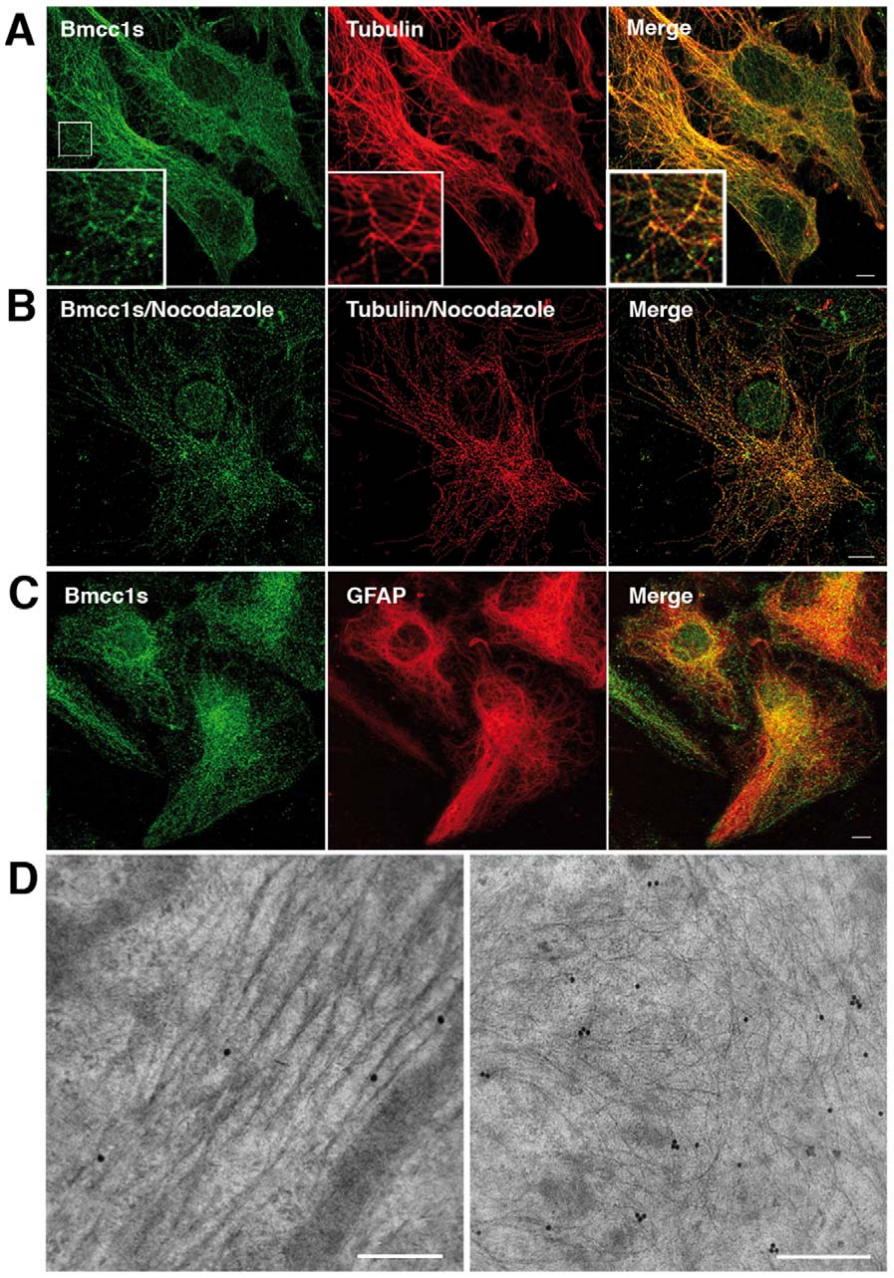
Subcellular localization of Bmcc1s in primary cultures of astrocyte. (A–C) Confocal section images of primary astrocytes immunostained for endogenous Bmcc1s (green) and α-tubulin or GFAP (red). Merge images showed that Bmcc1s forms punctate spots mainly distributed along α-tubulin stained microtubules (A) and partially colocalized with GFAP-positive intermediate filaments (C). Boxed regions in A indicate the fields enlarged in each image. B. In nocodazole-treated primary astrocytes (10 µM, 1 h), Bmcc1s followed the disrupted α-tubulin microtubular staining. (D) Immunogold labelling and electron mircroscopy analysis of primary astrocytes showed that Bmcc1s localized on cytoskeleton-type structures compatible with microtubules (left) and intermediate filaments (right). Bars: 10 µm (A–C); 200 nm (D).

**Figure 4:**
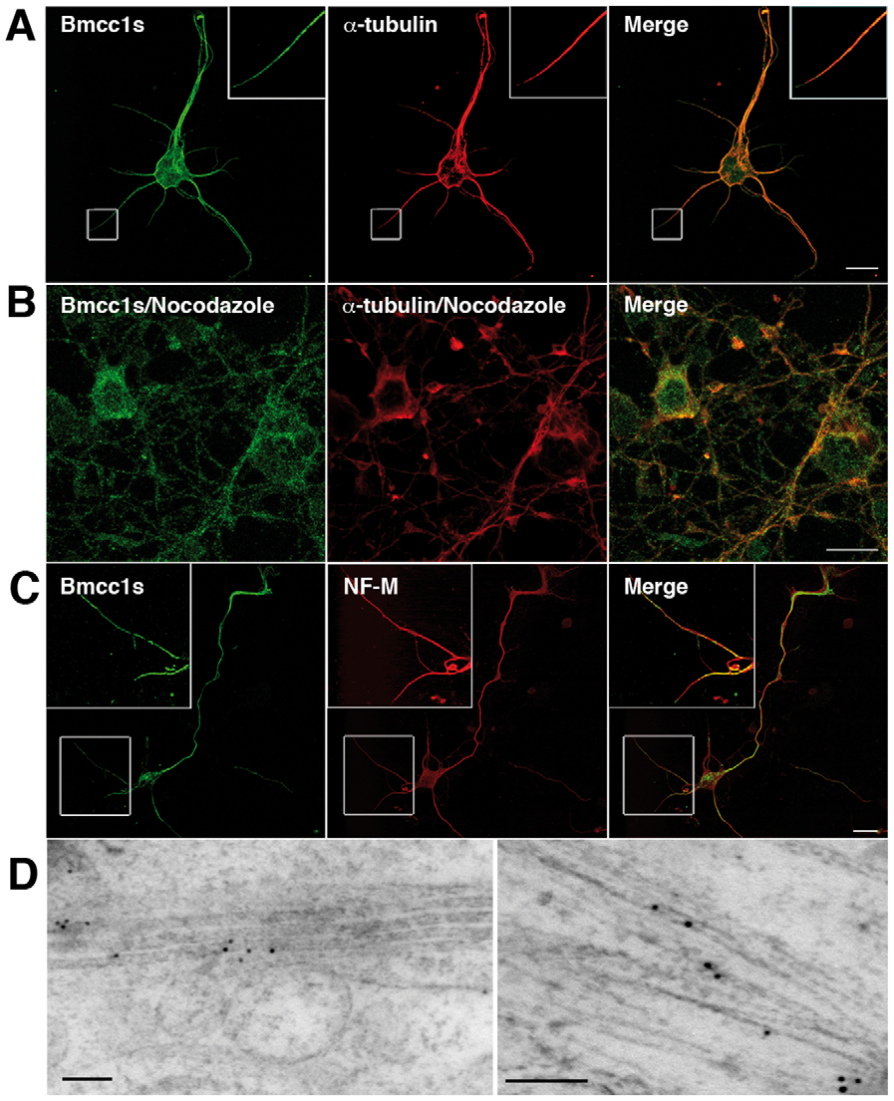
Subcellular localization of Bmcc1s in primary neurons. (A–C) Confocal section images of primary neurons after 7 days of culture immunostained for endogenous Bmcc1s (green) and α-tubulin or neurofilament subunit M (NF-M) (red). Merge images showed that Bmcc1s colocalizes with α-tubulin (A) and NF-M (C) immunoreactivity signal. Boxed regions in A and C indicate the fields enlarged in each image. B. In nocodazole-treated primary neurons (10 µM, 1 h), Bmcc1s followed the disrupted α-tubulin microtubular staining. (D) Immunogold labeling and electron microscopy analysis of primary neurons showed that Bmcc1s localized on cytoskeleton-type structures compatible with microtubules (left) and intermediate filaments (right). Bars: 10 µm (A–C); 100 nm (D).

**Figure 5:**
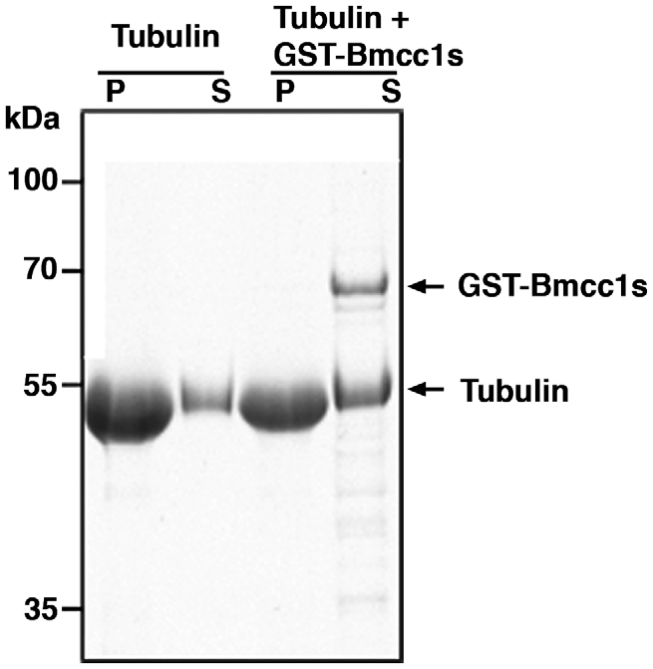
Microtubule Co-sedimentation assays. Taxol stabilized microtubules were incubated with or without GST-Bmcc1s. The samples were then sedimented through a 60% glycerol cushion. The supernatants (S) and pellets (P) were separated by SDS-PAGE and stained with Coomassie Blue. Tubulin (50 kDa) was mostly present in the pellet fraction with or without Bmcc1s, while Bmcc1s was only detectable in the supernatant.

Subcellular localization of endogenous Bmcc1s was analyzed in primary cultures of DIV 7 astrocytes and DIV 7 neurons. In immunofluorescence microscopy, Bmcc1s formed punctuate spots aligned in a filamentous fashion in astrocytes (Fig. 3A) while it was denser in neurons (Fig. 4A). Colabeling with α-tubulin demonstrated a colocalization of Bmcc1s with microtubules (in neurons: cell body 25±20% (n = 3); neurites 80±9% (n = 3); in astrocytes: 47±13% (n = 4)) (Figs. 3A, 4A). Incubation of both astrocytes and neurons with nocodazole resulted in a partial depolymerization of microtubules and a parallel displacement of α-tubulin and Bmcc1s, strongly supporting the association of Bmcc1s with microtubules (Figs. 3B, 4B). Colabeling experiments also revealed that part of the Bmcc1s signal colocalized with GFAP (Glial Fibrillary Acidic Protein), the astrocyte-specific intermediate filament protein (57±16% (n = 4)) (Fig. 3C), and with NF-M, a component of the intermediate filaments in neurons (cell body 19±14% (n = 3); neurites 64±12% (n = 3)) (Fig. 4C). Consistently, immunoelectron transmission microscopy detected endogenous Bmcc1s on cytoskeleton-type structures compatible with microtubules and intermediate filaments (Figs. 3D, 4D). In order to further explore the relationship between Bmcc1s and microtubules, we next examined the possibility of a direct binding of Bmcc1s to microtubules by standard microtubule binding *in vitro* assays (Fig. 5). Bmcc1s remained in the soluble fraction and did not co-sediment with taxol-stabilized microtubules. Thus, Bmcc1s colocalizes with microtubules and intermediate filaments, but *in vitro* it does not behave as a microtubule-binding protein.

### Bmcc1s interacts with the microtubule-associated protein MAP6

**Figure 6:**
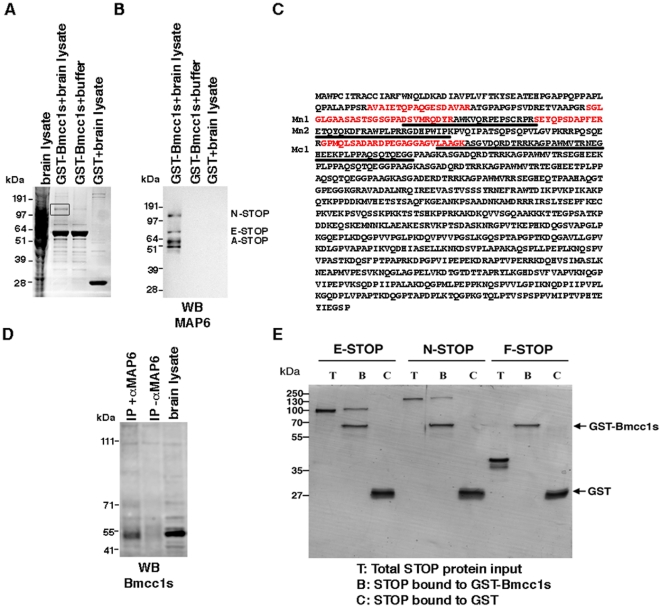
Bmcc1s interacts with MAP6. (A) GST-Bmcc1s or GST immobilized on glutathione sepharose beads were incubated with either a lysis buffer or a mouse brain lysate. After elution, bound proteins were resolved on SDS-PAGE in parallel with the mouse brain lysate, and visualized by Coomassie staining. A unique band (square) was analyzed by MALDI-TOF, where MAP6 was identified. (B) The presence of MAP6 and the specificity of its interaction with Bmcc1s were confirmed by Western blot of the GST eluates with 23N, a polyclonal anti-MAP6 antibody. Several bands corresponding to the neuronal MAP6 isoforms N-STOP (120 kDa) and E-STOP (80 kDa), the astrocyte MAP6 isoform A-STOP (60 KDa) and a 48 kDa isoform described in total brain protein extracts were revealed. (C) MALDI-TOF analysis revealed the presence of 4 peptides (in red) corresponding to MAP6. The microtubule-stabilizing modules Mn1, Mn2 and Mc1 of MAP6 are underlined. (D) Co-immunoprecipitation of MAP6 and Bmcc1s was performed using the 175 monoclonal anti-MAP6 antibody (IP+αMAP6), or no antibody (IP-αMAP6) as control, on mouse brain lysates. Precipitates were analyzed by Western blotting with Bmcc1 antiserum, in parallel with the mouse brain lysate. Bmcc1s was co-immunoprecipitated with MAP6. (E) Pull-down experiments of purified MAP6 isoforms: neuronal, N- and E-STOP and the fibroblast F-STOP, by purified glutathione-S-transferase (GST)-Bmcc1s or GST. Bound proteins were resolved on SDS-PAGE and Coomassie stained. N- and E-STOP were specifically retained by GST-Bmcc1s.

To further explore the functions of Bmcc1s, we searched for its binding partners by performing GST pull-down assays and matrix-assisted laser desorption/ionization time of flight (MALDI-TOF) mass spectrometry. Whole adult mouse brain lysates were incubated on either immobilized GST-Bmcc1s or on GST alone expressed and purified from *E. coli*. As controls, GST-Bmcc1s and GST were incubated with lysis buffer only. Bound proteins were eluted and resolved by SDS-PAGE. Visualization by Coomassie staining revealed a band around 120 kDa in the brain lysate retained by GST-Bmcc1s which was not present in controls (Fig. 6A). This band was subjected to trypsin-digestion followed by MALDI-TOF analysis and was identified as the microtubule-associated protein MAP6, also called STOP (Fig. 6C). MAP6 displays multiple isoforms which associate to microtubules and induce their stabilization [18]. In particular, they protect microtubules from depolymerization when cells are submitted to cold [19]. Here, MAP6 peptides sorted by mass spectrometry covered the N-terminal region of the neuronal MAP6 isoforms N-STOP and E-STOP (Fig. 6C). We next probed a Western blot of the eluates with the MAP6 polyclonal purified antibody 23N [19] (Fig. 6B). No signal was observed either in the GST-Bmcc1s/lysis buffer or in GST/brain lysate eluates, indicating the specificity of the Bmcc1s-MAP6 interaction (Fig. 6B). Several MAP6 isoforms were revealed in the GST-Bmcc1s/brain lysate eluate, namely the neuronal isoforms N-STOP (120 kDa) and E-STOP (80 kDa), the astrocyte isoform A-STOP (60 KDa) and a fibroblastic and astroglial 48 kDa isoform [18], [20]. In contrast, the main MAP6 fibroblast isoform F-STOP (42 kDa), also weakly expressed in astrocytes and neurons, was not detected. To confirm that *in vivo* MAP6 is a *bona fide* Bmcc1s-interacting partner, we immunoprecipitated endogenous MAP6 proteins from mouse brain lysates (Fig. 6D). Western blot analysis of the precipitate revealed the presence of Bmcc1s, supporting the fact that MAP6 and Bmcc1s are part of the same physiological complex in the brain. A shorter Bmcc1 isoform around 40 kDa was also co-immunoprecipitated indicating the possible interaction of MAP6 with other Bmcc1 isoforms in the brain. To further explore the interaction between Bmcc1s and MAP6, we next performed Bmcc1s GST pull-down assays using *in vitro* purified MAP6 isoforms (Material and methods). As shown in figure 6E, Bmcc1s specifically retained the neuronal MAP6 isoforms N-STOP and E-STOP. In contrast, in the same ionic conditions, the fibroblast MAP6 isoform F-STOP did not bind to Bmcc1s, as shown above (Fig. 6B). Finally, in agreement with these results, immunocytofluorescence experiments on primary neurons showed that Bmcc1s partially colocalized with endogeneous N-STOP (Fig. S6). Altogether, these results identify Bmcc1s as a new ligand of astroglial and neuronal MAP6 isoforms.

### Bmcc1s inhibits microtubule cold stability

**Figure 7:**
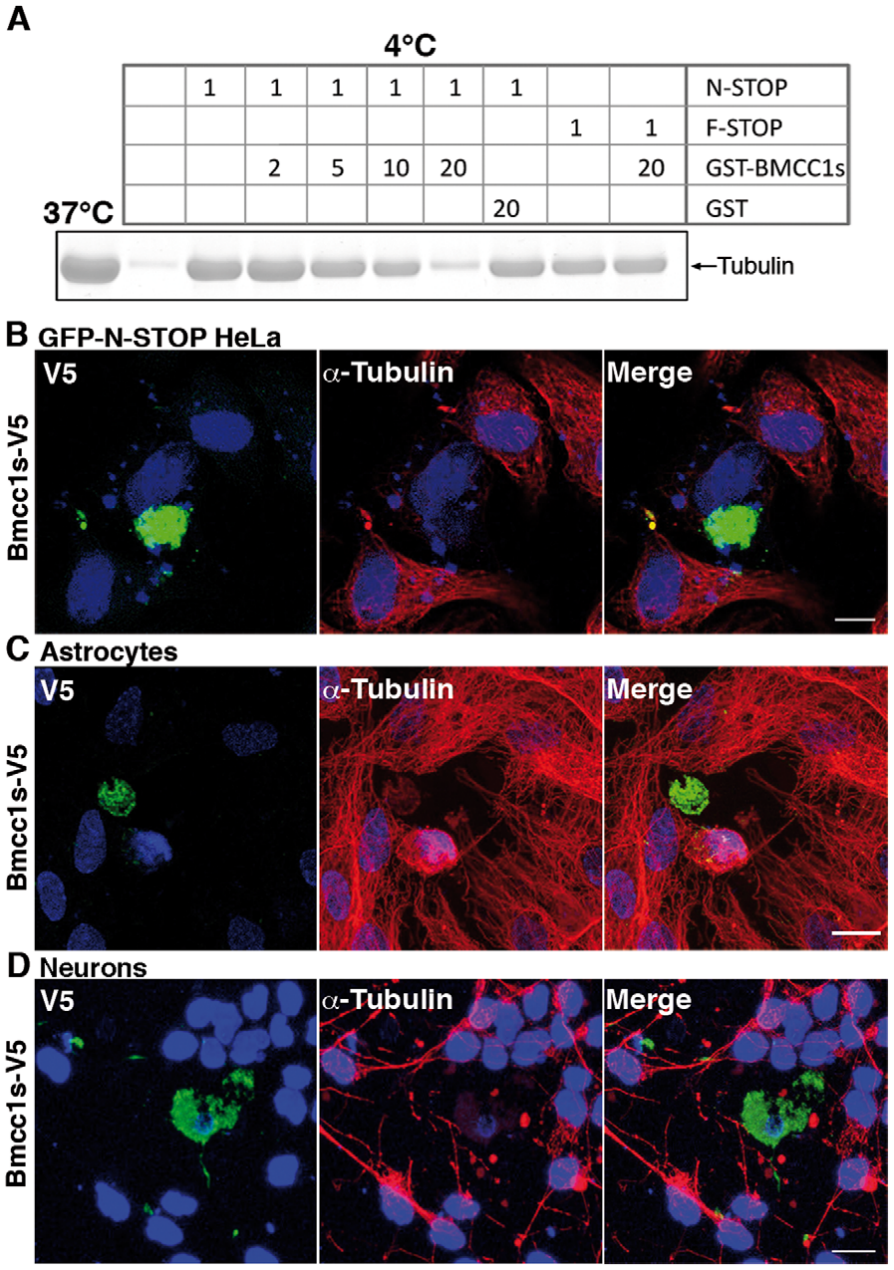
Bmcc1s inhibits the MAP6-induced microtubule cold stability. (**A**) Inhibition of N-STOP-induced microtubule cold stability by Bmcc1s *in vitro*. Microtubules polymerized at 37°C and subjected to cold were recovered by sedimentation and analyzed by SDS-PAGE and coomassie staining. The observed 50 kDa band corresponds to polymerized tubulin. At 4°C, almost no microtubules could be recovered. In contrast, they were preserved at 4°C in presence of N-STOP or F-STOP. Adding increasing concentrations of GST-Bmcc1s progressively decreased the level of microtubules in presence of N-STOP, but not of F-STOP. In contrast, GST alone had no effect. Numbers indicate the final concentration of the proteins in micromolar in the depolymerization reaction mix. Concentration of tubulin was 30 µM. (**B,C,D**) Confocal microscopy image projections of cells transiently transfected with a plasmid expressing Bmcc1s-V5. Twenty-four hours after transfection, cells were exposed to 0°C for 45 minutes. Following free tubulin extraction by cell permeabilization, cells were fixed and double-stained for α-tubulin antibody (red), and V5 (green). Nuclei were stained with DAPI (blue). (B) HeLa cells stably transfected with GFP-N-STOP; (C) Primary culture of astrocytes; (D) Primary culture of neurons. In Bmcc1s-V5 transfected cells (green), α-tubulin staining was almost gone and V5 staining either retracted in a ball shape in the case of GFP-N-STOP HeLa cells and astrocytes, or filled the cell body in neurons. Bars: 10 µm.

To explore the functional significance of Bmcc1s-MAP6 interaction, we next tested whether Bmcc1s could modulate MAP6-induced microtubule cold stability. *In vitro* polymerized microtubules at 37°C or subjected to cold were recovered by sedimentation and analyzed by SDS-PAGE and coomassie staining (Fig. 7A). At 4°C, almost no microtubules could be recovered. In contrast, they were preserved at 4°C in presence of N-STOP or F-STOP, demonstrating the microtubule-stabilizing effect of MAP6. Adding increasing concentrations of GST-Bmcc1s progressively lowered the level of microtubules in presence of N-STOP. In contrast, in presence of F-STOP, with which it does not interact, Bmcc1s had no effect, and neither did GST alone. Thus, Bmcc1s inhibited the N-STOP-induced microtubule cold stability *in vitro* without affecting that of F-STOP. We next assessed this effect in cultured cells. Transfection of N-STOP in HeLa cells, which are naturally devoid of MAP6, induces microtubule cold stability [21]. HeLa cells stably transfected with GFP-N-STOP (GFP-N-STOP HeLa) were transfected with a Bmcc1s-V5 expressing plasmid. Twenty-four hours following transfection, cells were placed at 0°C for 45 min and microtubule resistance to cold was assessed by α-tubulin immunostaining following free tubulin extraction (Material and methods) (Fig. 7B). In Bmcc1s-V5 transfected GFP-N-STOP HeLa cells, V5 staining entirely retracted to adopt a ball shape. In addition, α-tubulin staining was no longer detectable, indicating a complete depolymerization of microtubules compared to untransfected cells. We next tested whether Bmcc1s could have the same effect on endogenous MAP6 in primary cultures of astrocytes and neurons. Cells were transfected with Bmcc1-V5 and cold-treated in the next 24 h. After cold treatment, little or no α-tubulin staining could be seen in Bmcc1s-V5 expressing astrocytes. In addition, V5 staining entirely retracted to adopt a ball shape, as already observed in HeLa cells (Fig. 7C). Finally, Bmcc1s-V5-transfected neurons exposed to cold also lost α-tubulin staining (Fig. 7D). Altogether these observations indicate that Bmcc1s overexpression inhibits MAP6-induced microtubule cold stability.

### Bmcc1s displaces the neuronal MAP6 isoform N-STOP away from microtubules and induces the formation of membrane protrusions

**Figure 8:**
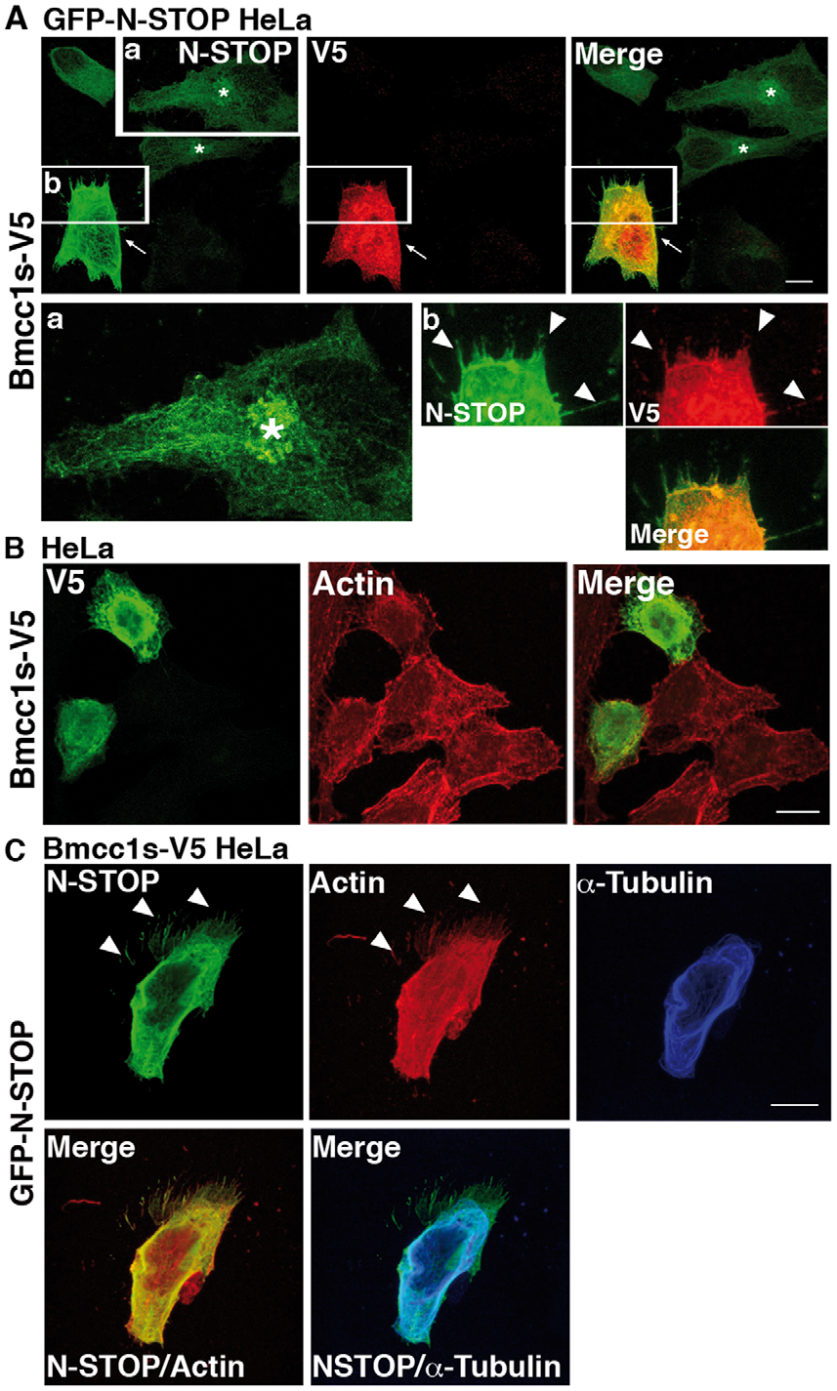
Bmcc1s overexpression displaces MAP6 away from the microtubules and induces the formation of membrane protrusions. (A) HeLa cells stably transfected with GFP-N-STOP (GFP-N-STOP HeLa) transiently transfected with an expression plasmid for Bmcc1s-V5. Twenty-four hours after transfection, cells were fixed and double-stained for N-STOP using the 23N polyclonal MAP6 antibody (green) and for Bmcc1s-V5 using a monoclonal anti V5 antibody (red). In untransfected GFP-N-STOP HeLa, N-STOP staining showed a microtubule-like pattern. The Golgi apparatus was also labeled (asterisks). Insert (a) is an enlargement of the squared region showing N-STOP staining in more detail. In the Bmcc1s-V5 GFP-N-STOP HeLa transfected cell (white arrows), N-STOP labeling became brighter, no longer featuring its typical microtubule-type distribution, and numerous membrane protrusions (white arrowheads) labeled for both V5 and N-STOP were seen. Insert (b) is an enlargement of the Bmcc1s-V5 GFP-N-STOP HeLa transfected cell. (B) Confocal microscopy images of HeLa cells transiently transfected with an expression plasmid for Bmcc1s-V5. Twenty-four hours after transfection, cells were fixed and double-stained for Bmcc1s-V5 using a monoclonal anti V5 antibody (green) and for F-actin using TRITC-conjugated phalloidin (red). (C) Confocal microscopy images of a Bmcc1s-V5 stably transfected HeLa cell (Bmcc1s-V5 HeLa) transiently transfected with an expression plasmid for GFP-N-STOP. Twenty-four hours after transfection, cells were fixed and stained for N-STOP using the 23N polyclonal anti-MAP6 antibody (green), for microtubules using a α-tubulin antibody (blue), and for F-actin using TRITC-conjugated phalloidin (red). Merge images show that N-STOP partially loses its microtubular staining, being more diffuse in the cell, and located in actin-rich membrane protrusions (white arrowheads). Bars: 10 µm.

By which mechanism **does** Bmcc1s inhibit the microtubule cold-stabilizing effect of MAP6? Since this phenomenon depends on the direct interaction of MAP6 with microtubules [22], we examined the subcellular distribution of N-STOP in GFP-N-STOP HeLa cells transfected or not with the Bmcc1s-V5 expressing plasmid. In GFP-N-STOP HeLa cells, N-STOP displayed a fibrillar aspect reminiscent of its association with microtubules (Fig. 8A) [18]. Accumulation of N-STOP staining in a perinuclear area possibly corresponding to the Golgi apparatus was also detected as previously described [23]. When Bmcc1s-V5 was transfected in these cells, the N-STOP labeling changed dramatically (Fig. 8A). It appeared brighter and part of it completely lost its cytoskeleton-type distribution, being more diffuse and concentrated at the cell periphery. Surprisingly, in contrast to untransfected GFP-N-STOP HeLa cells (Fig. 8A) or HeLa cells transfected only with Bmcc1s-V5 (Fig. 8B), GFP-N-STOP HeLa cells expressing Bmcc1s-V5 showed numerous membrane protrusions sprouting up in all directions and densely labeled for both V5 and N-STOP (Fig. 8A). In order to observe in parallel N-STOP and the actin and microtubule cytoskeletons, HeLa cells stably transfected with the Bmcc1s-V5 expressing plasmid (Bmcc1s-V5 HeLa) were transfected with the GFP-N-STOP expressing plasmid (Fig. 8C). As demonstrated by double immunostaining of α-tubulin and N-STOP, part of the N-STOP staining no longer localized on microtubules. Instead, as observed above (Fig. 8A), it was diffuse and more concentrated in actin-rich phalloidin-labeled areas at the cell periphery and in membrane protrusions (Fig. 8C). Thus, Bmcc1s relocates N-STOP away from microtubules. In addition, in presence of both Bmcc1s and N-STOP, numerous membrane protrusions are formed.

### Morphological effects are induced by Bmcc1s together with MAP6

**Figure 9:**
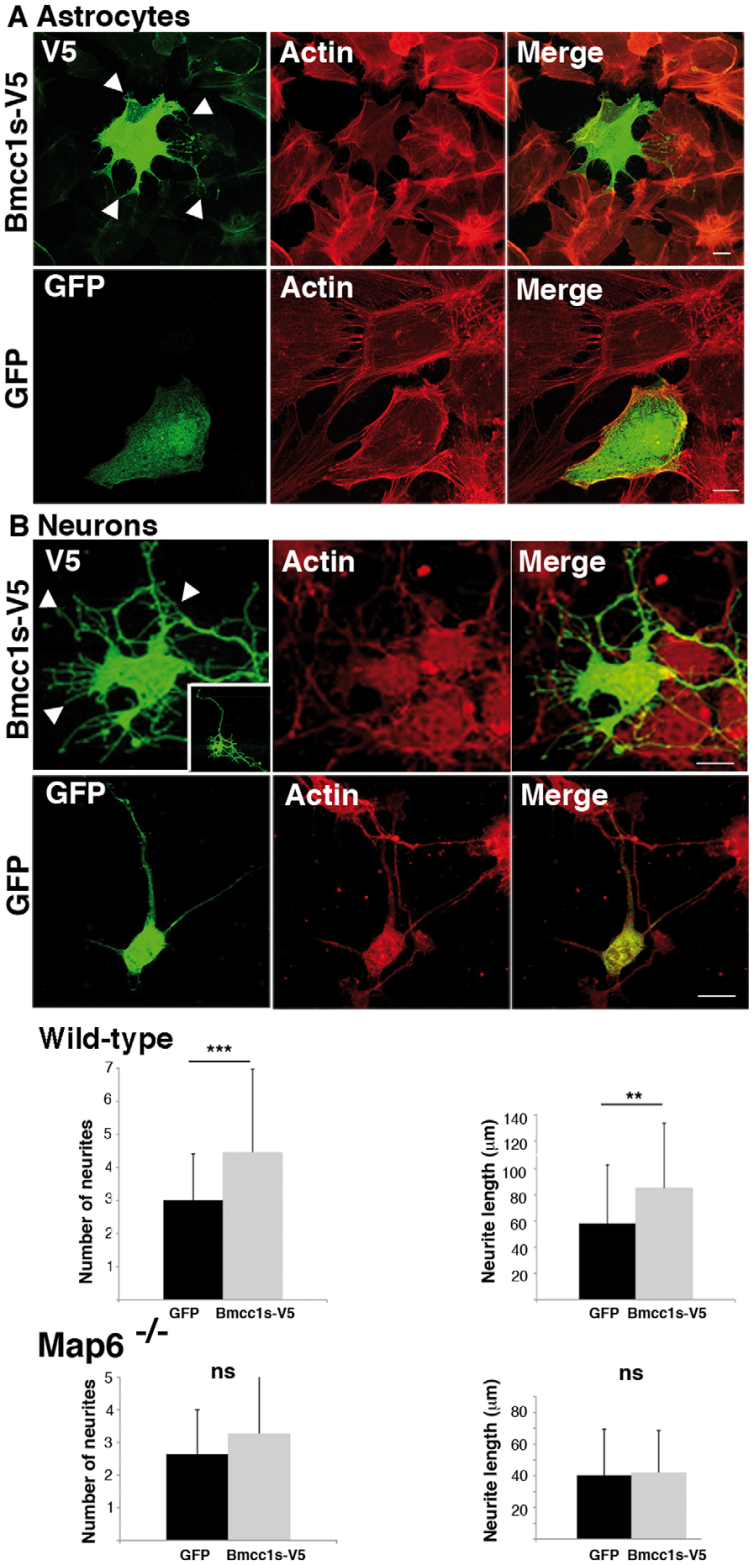
Morphological changes induced by Bmcc1s overexpression requires MAP6. Confocal microscopy image projections of cells transfected with a Bmcc1s-V5 or GFP expressing plasmid, and stained for V5 (green) and F-actin (detected with TRITC-conjugated phalloidin in red). Cells were fixed 24 h after transfection. (A) primary astrocytes; (B) primary neurons. The morphology of GFP-expressing cells (green) was unchanged compared to untransfected cells. In contrast, Bmcc1s-V5-expressing astrocytes and neurons developed numerous membrane protrusions (white arrowheads). Images in B illustrate representative confocal projections of the effect of Bmcc1s-V5 on neuritic growth and number in wild-type neurons. The whole Bmcc1s-V5 transfected neuron is shown in the insert. Histograms present means ± sd of the length of the longest neurite and of the number of neurites. *** p-value<0.0001 ** p-value<0.001. ns, not significant for 3 independent experiments using the two sample independent t-test. In neurons, length of the longest neurite, and number of neurites (or cell extensions starting from the soma) were significantly increased by Bmcc1s-V5 transfection, but not in MAP6-deleted neurons. Bars: 10 µm.

By which mechanism **do** Bmcc1s and MAP6 induce the formation of membrane protrusions? HeLa cells expressing only Bmcc1s-V5 or N-STOP did not show any obvious morphological change compared to HeLa expressing both proteins, indicating that the expression of one or the other is not sufficient to induce the formation of membrane protrusions. We therefore tested the effect of Bmcc1s transfection in cells expressing MAP6 endogenously. Primary astrocytes at DIV7 and neurons at DIV1 were transfected with the Bmcc1s-V5 expression plasmid. Cell morphology was analyzed 24 h after transfection. In agreement with our previous observations in HeLa cells expressing both N-STOP and Bmcc1s-V5 (Fig. 8A, 8C), in primary astrocytes Bmcc1s-V5 transfection resulted in the formation of long membrane protrusions sprouting out from the transfected cells in all directions, without obvious change in the pattern of actin stress fibers (Fig. 9A). No protrusion could be seen in cells transfected with a GFP-only expressing plasmid (Fig. 9A). Next, cell morphology as well as neurite length and number were compared in Bmcc1s-V5 and GFP-transfected primary neurons (Material and methods) (Fig. 9B). Compared to GFP, Bmcc1s-V5-expressing neurons often showed a very complex morphology, with an increase in the number of ramifications (Fig. 9B). In GFP-expressing neurons, the length of the longest neurite was 58.1±44.8 µm (n = 97), and the number of extensions starting from the soma was 3.0±1.4. In Bmcc1-V5 expressing neurons, the longest neurite reached 82.2±49.4 µm (n = 56), and the number of cell extensions was 4.4±2.5. These results indicated that Bmcc1s-V5 significantly increased neurite length (p-value<0.001) and number (p-value<0.0001). In contrast, actin labeling was comparable in GFP and Bmcc1s-V5-expressing neurons. We finally compared the morphology of Bmcc1s-V5 and GFP-transfected primary neurons prepared from MAP6 deficient mice [17] (Fig. 9B). Under Bmcc1s-V5 expression, the longest neurite reached 41.8±26.7 µm (n = 42), and the number of cell extensions was 3.3±1.8, whereas in GFP-transfected primary *Map6^−/−^* neurons, the longest neurite reached 40.2±29.3 µm (n = 88), and the number of cell extensions was 2.6±1.4. Thus, in *Map6^−/−^* primary neurons, Bmcc1-V5 transfection had no significant effect on neurite length and number (p-value>0.01). Altogether, these results suggest that Bmcc1s requires MAP6 as a cofactor to induce membrane protrusions.

## Discussion

In this study, we characterize Bmcc1s, a novel isoform of the BCH domain-containing molecule Bmcc1, predominantly expressed in the mouse brain. To date, BCH-proteins have been mostly studied for their capacity to bind and regulate the activity of Rho-small GTPases [1]–[9], with the exception of BNIP-H (or caytaxin), which also interacts with the kidney-type glutaminase [7], the peptidyl-prolyl isomerase Pin1 [10] and kinesin-1 [11]. Here we show that Bmcc1s interacts directly with MAP6, indicating that the functions of BCH-containing molecules are more diverse than initially expected. Bmcc1s binds to neuronal MAP6 isoforms N-STOP and E-STOP, astroglial MAP6 A-STOP and a 48 kDa astroglial and fibroblastic MAP6 isoform, but not to the fibroblastic F-STOP. Direct binding experiments with A-STOP and the 48 kDa isoforms were not performed since their sequences are not fully characterized. Nevertheless, our results indicated that interaction between MAP6 and Bmcc1s did not occur in domains shared by the neuronal MAP6 isoforms and F-STOP, i.e. all the central microtubule-stabilizing Mc modules and the microtubule-stabilizing Mn3 [18].

Bmcc1s localized on intermediate filaments and microtubules. Accordingly, our mass spectrometry analysis of the proteins pulled down by Bmcc1s not only demonstrated its interaction with the microtubule-associated protein MAP6 but also with the medium-sized neurofilament protein [24] (data not shown). The association of intermediate filaments to microtubules specifically involves detyrosinated microtubules (Glu-MTs) [25], [26], a subset of stable microtubules enriched in MAP6 [19]. Furthermore, MAP6 has been shown to co-aggregate with intermediate filaments in neurons [27]. Thus, interaction of Bmcc1s with MAP6 and its colocalization with microtubules and intermediate filaments may indicate a role for Bmcc1s in the cross-talk between both cytoskeletons.

The MAP6-induced microtubule protective effect operates through its direct interaction with microtubules [22]. Here, we showed that Bmcc1s inhibited the microtubule cold resistance and moved N-STOP away from microtubules. Microtubules consistently lost their resistance to the cold. This effect was also observed in primary astrocytes and neurons transfected with Bmcc1s, suggesting that endogenous MAP6 no longer interacted with microtubules. Thus, our results strongly suggest that Bmcc1s, by its ability to associate with MAP6, regulates MAP6-microtubules interaction and disrupts it when increased, leading to the loss of cold resistance. Therefore, Bmcc1s is a new molecular partner of MAP6 with the capacity to influence its microtubule interaction and protective functions.

Transfection of Bmcc1s also resulted in numerous membrane protrusions in astrocytes as well as in neurons, in which the length of the principal neurites was also increased. In neurons, these effects were abolished in absence of MAP6. In HeLa cells, the protruding effect was not observed unless Bmcc1s was co-expressed with N-STOP. Altogether, these results strongly suggest the requirement of both MAP6 and Bmcc1s to induce morphological alterations. What is the mechanism involved? In HeLa cells coexpressing Bmcc1s and N-STOP, N-STOP appeared partially relocated from the microtubules, in particular in actin-rich protrusions. Interestingly, MAP6 not only interacts with microtubules but with polymerized actin *in vitro*, suggesting that it may play a role in actin cytoskeleton dynamics [28]. Thus, an attractive hypothesis would be that, in the presence of high level of Bmcc1s, MAP6 dissociates from microtubules and binds to actin. In differentiating neurons, N-STOP when phosphorylated was shown to colocalize with actin rich areas in spikes and at branching points, but not with microtubules [28]. Thus, we tested whether such mechanism could occur under Bmcc1s overexpression in protein extracts prepared from Bmcc1s and N-STOP transfected HeLa cells. The molecular weight of N-STOP did not shift in presence of Bmcc1s (data not shown), suggesting that Bmcc1s does not influence the phosphorylation status of N-STOP. *In vitro*, the effect of Bmcc1s on the N-STOP microtubule stabilizing effect appeared to be dose-dependent, thus an alternative hypothesis would be that increased levels of Bmcc1s reduce the level of MAP6 available for microtubule interaction, favoring its link to actin. The question now arises as to how MAP6 could possibly act on actin dynamics to induce the formation of membrane protrusions, which is still an open issue.

In conclusion, Bmcc1s is a novel Bmcc1 isoform predominantly expressed in the brain and present in astrocytes and neurons. It localizes on intermediate filaments and microtubules and directly interacts with the microtubule-associated protein MAP6. Overexpression of Bmcc1s displaces MAP6 from microtubules, inhibiting its protective effect to cold and affecting cell morphology. The transcription of *BMCC1* has been found to be strongly upregulated in the neurodegenerative Rett syndrome [16]. Thus, the alteration of astroglial and neuronal cell morphology and the modulation of MAP6 functions, which result from Bmcc1s upregulation, could represent molecular and cellular mechanisms involved in this pathology.

## Materials and Methods

### Animal experimentation

In compliance with the European Community Council Directive of November 24, 1986 (86/609/EEC), research involving animals has been authorized by the Direction Départementale de la Protection des Populations, Préfecture de l'Isère, France (permit n°38 09 18). Every effort has been made to minimize the number of animals used and their suffering. This study has been approved by the local ethics committee of Grenoble Institut des Neurosciences.

### Data deposition

The sequence of Bmcc1s reported in this paper has been deposited in the EMBL/GenBank/DDBJ databases: accession number FR69337.

### Antibodies

Polyclonal antibodies against a mixture of two synthetic peptides of mouse Bmcc1s were raised in rabbit (Covalab). Peptide sequences were: (24–33) SLDLNGSHPR, and (101–113) SIPEYTAEEERED. Numbering refers to EMBL accession number FR69337. To test the specificity of the antibodies, 1 µl of rabbit serum was incubated overnight at 4°C with *in vitro* synthesized GST-Bmcc1s bound to glutathione-sepharose beads. Eluates were used to probe Western-blots of mouse brain protein extracts (data not shown). Primary antibodies used were: Anti-MAP6 polyclonal 23N [19] (dilution 1∶1000) and Monoclonal 175 [29], polyclonal and monoclonal anti-V5 (Sigma-Aldrich) (dilution 1∶500), monoclonal anti α-tubulin (Sigma-Aldrich) (dilution 1∶1000), polyclonal anti-RhoA (Santa Cruz Biotechnology) (dilution 1∶1000), horseradish-peroxidase-conjugated monoclonal anti-GAPDH (Sigma) (dilution 1∶2500). Monoclonal antibody M20 against NF-M was kindly provided by Dr Beat M. Riederer (Hornung et al., 1999) (dilution 1∶10). Secondary antibodies used were: Alexa-conjugated goat anti-mouse and anti-rabbit IgGs (Molecular probes) (dilution 1∶2000), Horseradish-peroxidase-conjugated goat anti-mouse and anti-rabbit antibodies (Amersham) (dilution 1∶2500).

### 5′ RACE PCR RT-PCR and cloning

Elongation of Bmcc1s cDNA 5′ end was performed using an adult mouse brain cDNA library generated by the Marathon method (Clontech). The reverse oligonucleotides used for the specific amplification of Bmcc1 were: (first experiment) (3971–3990 in exon 21) 5′-AGGGCTGTGCAGAACCATGA -3′, (second experiment) (398–422 in exons 11 and 12) 5′-TGGCCGTGGGATCTTCATGGTTAGT -3′, and (third experiment) (1185–1209 in exon 21) 5′-GTGGAGATGTCACCATCCCTGTTGC-3′. Elongation time was 5 min, using the expand High fidelity Taq polymerase (Roche). PCR products were cloned into pCR2.1-TOPO (Invitrogen) and sequenced. For RT-PCR, total RNA was extracted from adult mouse tissues using the RNeasy lipid tissue kit (Qiagen). Reverse transcription was performed on 1 µg of total RNA using Superscript II reverse transcriptase (Invitrogen). PCR was performed using the 5′ Race primer (first experiment) (3971–3990 in exon 21) and the upstream forward primer (3895–3950) 5′-CCCCTAGGGCATACCGATGA-3′. Control Hprt amplification was performed using the following primers: Hprt.f 5′ GTTGGATACAGGCCAGACTTTGTTG 3′; Hprt.r 5′ GATTCAACTTGCGCTCATCTTAGGC 3′. Expression construct for Bmcc1s was performed by RT-PCR on adult mouse brain mRNA using the Superscript II reverse transcriptase (Invitrogen) and the LA-Taq polymerase (Takara), with the following primers: (146–170 in exon 10) 5′-GGAATGGATATCCACTTCGAGGAGG-3′ and (1093–1117 in exon 21) 5′-CGGCTTCTCCTTCAGCTTCATGTCA-3′. Amplification product was cloned into pcDNA3.1/V5-His-TOPO (Invitrogen). glutathioneS-transferase (GST)-Bmcc1s was cloned in pGEX-4T1 (Amersham). For this construct, Bmcc1s was amplified using primers containing EcoR I or Sal I restriction sites (in italics): (149–163 in exon 10 underlined) 5′-GG*GAATTC* ATGGATATCCACTTC_3′ and (1106–1121 in exon 21) 5′-GG*GTCGAC* GCTACGGCTTCTCCTT-3′. Each construct was verified by sequencing on both strands. The pSG5-N-STOP expressing plasmid carried rat N-STOP cDNA into the Bgl II site of pEGFP-C1 (Clontech) [21]. pEGFP-N-STOP plasmid was generated by subcloning the Bgl II fragment of pSG5-N-STOP. All numberings for primers refer to EMBL accession number FR69337.

### Protein extraction from mouse tissues and Western blotting

Adult mouse tissues were dissected and reduced in powder at −80°C, immediately dissolved in PBS with 2% SDS, and 1× EDTA-free Complete Protease Inhibitor (Roche). Lysates were sonicated twice at 10 Hz (Vibra cell VCX130) and centrifuged 20 min at 10000 g at 4°C. Supernatants were boiled in 5× Laemmli loading buffer. Protein content was measured using the Pierce 660 nm protein assay reagent (Thermo scientific). Equal amounts of proteins were separated by denaturing electrophoresis in NuPAGE 3–8% Tris acetate gradient gel (Invitrogen), electrotransfered to nitrocellulose membranes, first analyzed using the Bmcc1 antiserum and HRP-congugated rabbit antibodies, and then using a GAPDH-HRP coupled antibody. HRP activity was visualized by ECL using Western Lightning plus enhanced chemoluminescence system (Perkin Elmer). Chemoluminescence imaging was performed on a LAS4000 (Fujifilm). GAPDH expression was used as a loading reference.

### Cell culture, transfection and drug treatments

Primary cortical neurons were prepared at embryonic day 15 as previously described [30]. Primary cultures of cortical astrocytes were prepared at post-natal day 2 as previously described [31]. HeLa cells (ATCC CCL-2) were grown in Dulbecco's-modified Eagle medium (DMEM; Invitrogen) supplemented with 10% fetal calf serum, in 5% CO_2_ at 37°C. Stable transfectants for pEGFP-N-STOP were selected with 500 µg/ml G418 during 15 days. After 7 days of culture without G418, cells were then maintained with 250 µg/ml G418 and FACS-sorted. Stable transfectants for Bmcc1s-V5 were selected by adding 500 µg/ml G418. Transient transfections were carried out on HeLa or DIV 7 primary astrocytes with Lipofectamine 2000 according to the manufacturer's instructions (Invitrogen). Primary Neurons were transfected at DIV1. In this case, cells were incubated only 30 min with the lipofectamine plasmid mix and returned to their initial culture medium. Estimation of the neurite length and number was done 24 h after transfection using ImageJ software. Statistical analysis of the results was done using the T-test. Three independent experiments were performed. For the microtubule cold stability analysis, cells were exposed 45 min to the cold on ice, permeabilized in lysis buffer (30 mM Pipes, 1 mM EGTA, 1 mM MgCl_2_, 10% glycerol, 1% Triton X-100, pH 6.75) for 1 min and processed for immunofluorescence [23]. Control experiments were performed using the pmaxGFP plasmid (Lonza). Microtubule depolymerizing treatment was performed using 10 µM Nocodazole for 1 h at 37°C.

### Immunocytofluorescence

Cells were grown on coverslips, fixed in 4% phosphate-buffered (PBS) paraformaldehyde (PFA) for 10 min at room temperature, and processed for immunofluorescence as described [32]. See the “antibodies” section for the dilutions of the various antibodies. Fluorescence images were taken in a SP5 confocal microscope (Leica). F-actin was detected with TRITC-conjugated phalloidin (Sigma). Colocalization of Bmcc1s with α-tubulin, GFAP and NF-M was estimated using the ImageJ software.

### Immnunoelectron microscopy

Astrocytes were fixed with 4% (w/v) PFA and 0.1% glutaraldehyde (Polysciences, Inc, Warrington) in PBS. After being washed in PBS, the cells were treated with ammonium chloride (0.13 g/50 ml of PBS) for 30 min at 4°C, gradually dehydrated in ethanol, and embedded in lowicryl Hm20 (Polysciences) in an AFS REICHERT (Leica) after a progressive lowering of temperature. After polymerisation under UV light at 45°C below zero for 48 h, pale yellow sections were incubated for 30 min in goat gold conjugate-blocking solution (Aurion, Wageningen, Netherlands). The sections were washed (three times for 5 min each) in PBS 0.1% bovine serum albumin-c (Aurion). Sections were subsequently incubated overnight at 4°C in the same buffer with Bmcc1 antiserum. After extensive washes (six times for 5 min each), the sections were incubated 1 h at room temperature in 20 nm gold-conjugated secondary antibodies against rabbit IgG (1∶50, British Biocell International, Cardiff, UK), washed (six times for 5 min each) in the incubation buffer, and then in PBS (two times for 5 min), followed by a 5 min fixation in 2% glutaraldehyde in PBS. After a wash of 5 min in PBS and six washes of 2 min in distilled water, sections were counterstained with uranyl acetate and lead citrate for inspection with a Philips tecnai 12 electron microscope (FEI the Eindhoven, The Netherlands). Primary neurons (11 DIV) were plated on thermanox coverslips (Nunc, Inc. Naperville, IL) and frozen in a Leica HPM 100 apparatus under a pressure of 2100 bar. After freezing, samples were rapidly transferred to liquid nitrogen. Cryosubstitution and embedding of the cells were then performed in a AFS 2 apparatus (Leica) in anhydrous methanol with 1,5% uranyl acetate at −90°c for 40 h with one change of solution. After extensive washes in anhydrous methanol, cells were slowly warmed to −45°C (5°C/h) and gradually embedded in lowicryl Hm20 (Polysciences). After polymerisation under a UV light at −45°C during 48 h, coverslips were warmed to room temperature, mounted on resin block and cut in parallel to the cell plan. The coverslips were completely removed with the glass knife until reaching the cells. Thin sections were cut using a Leica ultracut E and incubated 30 min in goat gold conjugates blocking solution (Aurion). Sections were then washed three times for 5 min in PBS with 0.1% bovine serum albumin-c (Aurion), and subsequently incubated overnight at 4°C in the same buffer with the Bmcc1 antiserum (1∶200). After six washes (5 min each), sections were incubated 1 h at room temperature in 10 nm gold-conjugated secondary antibodies against rabbit IgG (1∶50, British Biocell International) and washed six times 5 min in the incubation buffer, twice in PBS and 5 min fixation in 2% glutaraldehyde in PBS. After a 5 min PBS wash and six washes of 2 min in distilled water, sections were counterstained with uranyl acetate and lead citrate for inspection with a Philips tecnai 12 electron microscope (FEI the Eindhoven, The Netherlands).

### Sedimentation of Bmcc1s with Microtubules

All proteins were preclarified at 150,000 g for 15 min in a TL-100 Ultracentrifuge (Beckman) at 4°C before the experiment started. Microtubule-binding assay was performed as previously described, using 40 µg taxol-stabilized microtubules (4 µM) as substrates and 1 to 4.5 µg of purified GST-Bmcc1s [33].

### *In vitro* effect of Bmcc1s on microtubule stability

Microtubules were polymerized *in vitro* from bovine brain tubulin (60 µM) in 2 µl of PEM (Pipes 100 mM pH6.75, EGTA1 mM, MgCl_2_ 1 mM) containing 1 mM GTP at 37°C. After 45 min, microtubules were either preserved by dilution in 20 µl of 60% sucrose/PEM solution at 37°C or diluted with one volume (2 µl) of ice-cold PEM-T solution (PEM+0.1% Tween 20) containing 2 µM of N-STOP or F-STOP proteins alone or mixed with either 4, 10, 20, 40 µM of GST-Bmcc1s or 40 µM of GST and incubated for 30 min on ice. Reaction mixes were then diluted with 20 µl of 37°C solution of 60% sucrose in PEM, loaded on warm 60% sucrose in PEM cushions (80 µl) and centrifuged at 200 000 g for 30 min at 37°C. Pellets were briefly washed with 300 µl of PEM solution at 37°C, re-suspended in Laemmli buffer and analyzed by SDS-PAGE and coomassie staining.

### Identification of Bmcc1s-binding proteins

Adult mouse brains were homogenised in binding buffer (PBS with 5% glycerol, 5 mM MgCl_2_, 0.1% Triton X-100, and 1× EDTA-free Complete Protease Inhibitor (Roche)) with a Dounce tissue grinder. Lysates were sonicated twice at 10 Hz (Vibra cell VCX130). Triton X-100 concentration was adjusted to 1% and lysates were incubated 1 h at 4°C. After 20-min centrifugation at 10000 g at 4°C, protein content of the cleared lysates was measured using the BCA protein assay (Thermo scientific). 500 µg of proteins were incubated with GST-Bmcc1s fusion protein, bound to glutathione-sepharose beads overnight at 4°C. After five washes with binding buffer containing 150 mM NaCl, proteins were boiled in 5× Laemmli loading buffer, separated by denaturing electrophoresis in NuPAGE 4–12% SDS-polyacrylamide gradient gel (Invitrogen), and visualized by Coomassie staining (BioRad). For mass spectrometry (MS) analyses, gel slices were reduced, alkylated, and subjected to digestion with trypsin (Roche) as previously described [34]. The extracted peptides were dried and resolubilized in solvent A (95∶5 water/acetonitrile in 0.1% [wt/v] formic acid). The total digestion product of a gel slice was used for two liquid chromatography-tandem MS (LC-MS/MS) analyses (1/5 and 4/5). The extracted peptides were concentrated and separated on an HPLC system (Ultimate 3000; Dionex), coupled to the nano-electrospray ionization interface of a mass spectrometer (QSTAR Elite; Applied Biosystems) using a PicoTip emitter (10 µm in diameter; New Objectives). HPLC mobile phases contained solvent A and solvent B (20∶80 water/acetonitrile in 0.085% [wt/v] formic acid). Bound peptides were eluted with a gradient of 5–50% of solvent B. Information-dependent acquisition was used to acquire MS/MS data, with the experiments designed so that the three most abundant peptides were subjected to collision-induced dissociation, using nitrogen as collision gas. Data from the information-dependent acquisition experiments were searched twice using MASCOT (Matrix Science) and PHENYX (Geneva Bioinformatics) software on the NCBI nr Mus musculus database (National Library of Medicine, Bethesda, 2009 07 03, 143362 protein entries). All data were validated using myProMS [35].

### Co-immunoprecipitation

Adult mouse brains were homogenized in RIPA lysis buffer (50 mM TrisHCl pH 8.0, 150 mM NaCl, 1% NP-40, 0.5% sodium deoxycholate, 0.1% SDS, 1× EDTA-free Complete Protease Inhibitor (Roche)) with a Dounce tissue grinder. After 20 min centrifugation at 10000 g at 4°C, the lysate was precleared with protein G sepharose (GE Healthcare) and protein content was measured using the BCA protein assay (Thermo scientific). 5 µl of purified monoclonal 175 anti-MAP6 antibody was added to 500 µg of proteins and incubated at 4°C overnight. Immunocomplexes were captured by adding protein G sepharose for 1 hour at 4°C. After five washes with lysis buffer, beads were resuspended and boiled in 5× Laemmli loading buffer to release immunoprecipitates. Samples were separated by denaturing electrophoresis in NuPAGE 4–12% SDS-polyacrylamide gradient gel (Invitrogen), electrotransfered on nitrocellulose membranes, and analysed by Western blotting with Bmcc1 antiserum and HRP-congugated rabbit antibodies, using Western Lightning plus enhanced chemoluminescence system (Perkin Elmer).

### In vitro Bmcc1s-MAP6 interaction

MAP6 isoforms were expressed as N-terminal His-tagged proteins in High-five insect cells. The F-STOP protein was successively purified on anion-exchange Q-sepharose, Nickel-NTA and calmodulin-agarose columns. The N-STOP protein was purified by affinity first on a Ni-NTA column and then on a column carrying the monoclonal 175 antibody. N-STOP was eluted using the corresponding antigenic peptide. Regarding E-STOP, affinity purification on Ni-NTA beads was sufficient to obtain a protein with a high degree of purity. GST and GST-Bmcc1s were expressed in E. coli and purified on glutathione-agarose beads according to the manufacturer's instructions. All the proteins were extensively dialysed against PEM buffer (PIPES 100 mM pH6.6, EGTA 1 mM, MgCl_2_ 1 mM). For GST pull-down assays, 10 µl of glutathione-agarose beads were mixed with either 2 µg of GST-BMCCsh1 or 4 µg of GST and 1.5 µg of one of the purified STOP isoforms. After 2 h incubation at 4°C in 300 µl of incubation buffer (PEM buffer plus 50 mM KCl, 0.05% Triton-X100 and 1 mM DTT), the beads were sedimented at 300 g for 30 seconds, washed three times with 500 µl of incubation buffer and resuspended in Laemmli buffer. Samples were separated on a 12.5% SDS-PAGE and Coomassie stained.

## Supporting Information

Figure S1 **Mouse Bmcc1/Prune2 gene, transcripts and proteins.** (A) Schematic representation of mouse Bmcc1 gene. All exons and introns are at scale, unless indicated. Insert at exons 7a/7b indicates the orthologous exon 4 of the human PCA3 gene on the opposite strand, which overlaps with exon 7a. A & B. Exons are boxed, in black for the coding sequence and in white for the 5′ and 3′ non-coding sequences. Alternative start and stop codons are indicated. (B) Schematic representation of mouse Bmcc1 transcripts. Scale is as in A, and transcripts are given with their accession number, size, library type, and exon composition. Solid bar under exon 21 indicates DNA arrays probe set. Primers for 5′ RACE experiments are indicated by arrows under exons 11/12 and 21. Dashes indicate reading frames that are still open. (C) Schematic representation of mouse protein Bmcc1 protein isoforms encoded by the corresponding transcripts shown in B. Proteins are at scale, with their accession number, size, and library type. Corresponding coding exons are boxed in light gray. Dashes indicate that protein may be longer. Conserved domains described in [36] are indicated on the top of the longest protein, as the antigenic peptides (asterisks) used to generate the Bmcc1s antiserum. In comparison to the human sequences presented in figure S2, Bmcc1 displays an additional exon 7a, generating specific C-termini in the N-ter proteins. Second, translation of mouse C-ter Bmcc1 proteins is initiated at the ATG initiation codon within exon 10, while in human BMCC1 it starts either in exon 9b or in exon 9c. Consequently, all Bmcc1 C-ter proteins share the same N-terminus, which differs in human. Finally, exon 7a overlaps the orthologous human PCA3 exon 4 on the opposite strand, while human PCA3 coding-exons located in intron 6 do not overlap with BMCC1 exons [36].
(PDF)

Figure S2 **Human BMCC1/PRUNE2 gene, transcripts and proteins.** (A) Schematic representation of the BMCC1 gene. All indicated exons and introns are at scale. Insert at intron 6 indicates the four PCA3 gene exons on the opposite strand. (A,B) Exons are boxed in black for the coding sequence and in white for the 5′ and 3′ non-coding sequences. Alternative start and stop codons are indicated. B. Schematic representation of human BMCC1 transcripts. Scale is as in A, and transcripts are given with their accession number, size, library type, and exon composition. Dashes indicate still opened reading frames. C. Schematic representation of human BMCC1 protein isoforms encoded by the corresponding transcripts shown in B. Proteins are at scale, with their accession number, size, and library type. Corresponding coding exons are boxed in light gray. Dashes indicate that protein may be longer. Bmcc1-1 to Bmcc1-4 are described in [36]. Accession numbers of the partial transcripts (EST) linking exons 1–6 to the remaining exons, or demonstrating the presence of the ortholog of mouse exon 18 in human transcripts and gene are in italics. Conserved domains described in [36] are indicated at the top of the longest protein, as well as the conserved epitope (asterisk) used to generate Bmcc1 antiserum.
(PDF)

Figure S3 **Expression profile of Bmcc1s.** RT-PCR using total RNA extracted from various mouse tissues on the 3′ end of the Bmcc1s 3′UTR. Amplification occurred mainly in the brain, demonstrating that Bmcc1s expression is highly specific to this organ. Hprt amplification was used as an internal control.
(PDF)

Figure S4 **Specificity test of the Bmcc1s antiserum.** Immunoblotting of adult mouse cortex proteins with the Bmcc1s antiserum (Control), or the antiserum preincubated on sepahrose bound GST or increasing concentrations of sepharose bound GST-Bmcc1s. GAPDH expression is shown as a loading reference.
(PDF)

Figure S5 **Immunodetection of Bmcc1s in the post-natal developing brain.** Immunoblot of endogenous Bmcc1 isoforms in mouse brain lysates of post-natal day (P) 1 to 4 months, using Bmcc1 antiserum. A major 50 kDa band (arrow) corresponding to Bmcc1s was detected at all stages.
(PDF)

Figure S6 **Bmcc1s colocalizes with the neuronal MAP6 isoform N-STOP in primary neurons.** Confocal section images of primary neurons immunostained for Bmcc1s (green) and N-STOP (red) using the monoclonal antibody 175 [29]. Bar: 10 µm.
(PDF)
