# Supplementary material for: Bmcc1s, a Novel Brain-Isoform of Bmcc1, Affects Cell Morphology by Regulating MAP6/STOP Functions
Source: PLoS One. 2012 Apr 16;7(4):e35488. doi: 10.1371/journal.pone.0035488 (PMC3327665; doi:10.1371/journal.pone.0035488)

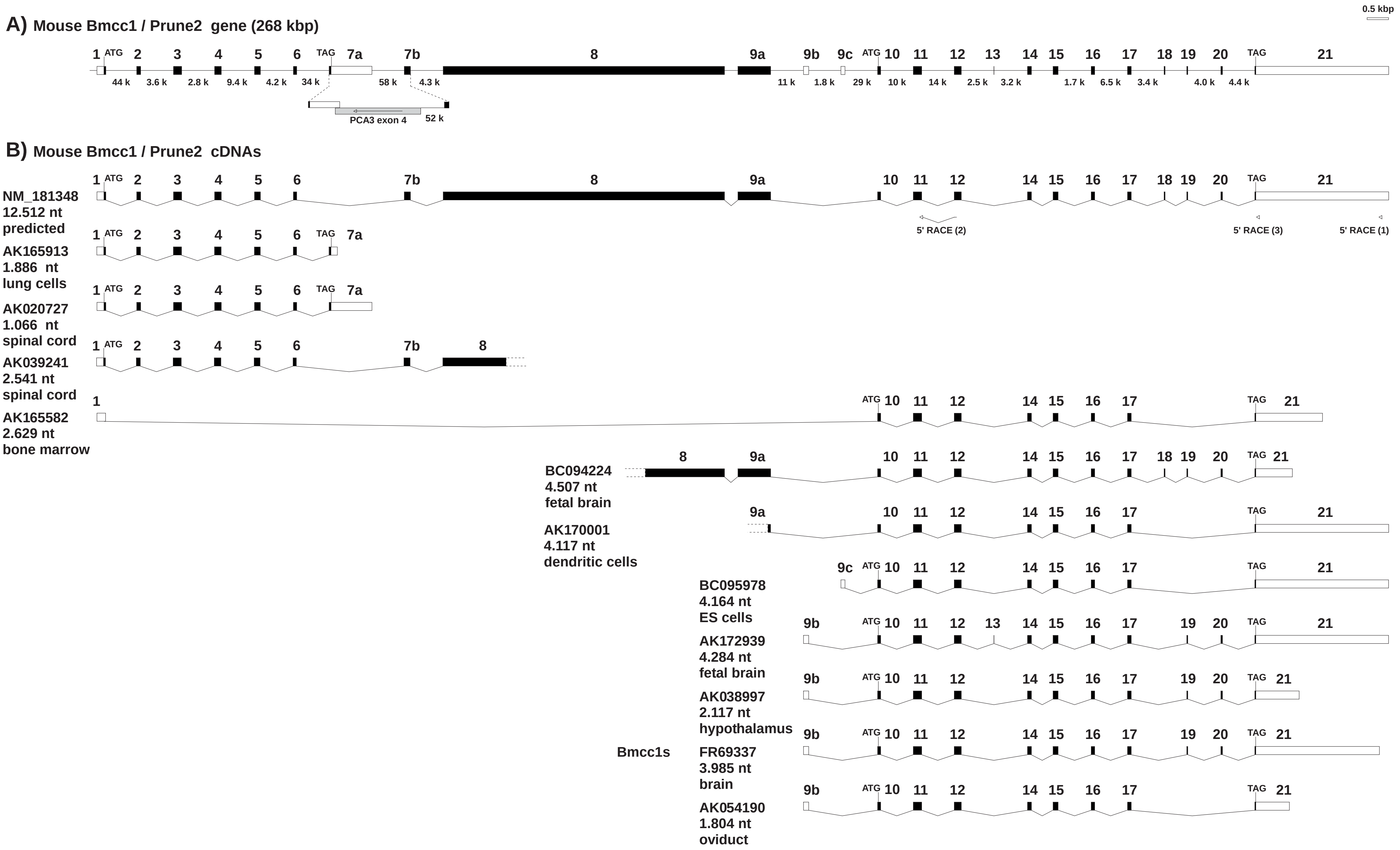
A) Mouse Bmcc1 / Prune2 gene (268 kbp)
0.5 kbp
1 ATG 2 3 4 5 6 TAG 7a 7b 8 9a 9b 9c ATG 10 11 12 13 14 15 16 17 18 19 20 TAG 21
44 k 3.6 k 2.8 k 9.4 k 4.2 k 34 k 58 k 4.3 k 11 k 1.8 k 29 k 10 k 14 k 2.5 k 3.2 k 1.7 k 6.5 k 3.4 k 4.0 k 4.4 k
PCA3 exon 4
52 k
B) Mouse Bmcc1 / Prune2 cDNAs
NM_181348
12.512 nt
predicted
5' RACE (2)
5' RACE (3)
5' RACE (1)
AK165913
1.886 nt
lung cells
AK020727
1.066 nt
spinal cord
AK039241
2.541 nt
spinal cord
AK165582
2.629 nt
bone marrow
BC094224
4.507 nt
fetal brain
AK170001
4.117 nt
dendritic cells
BC095978
4.164 nt
ES cells
AK172939
4.284 nt
fetal brain
AK038997
2.117 nt
hypothalamus
Bmcc1s
FR69337
3.985 nt
brain
AK054190
1.804 nt
oviduct

C) Mouse BMCC1 / Prune2 proteins

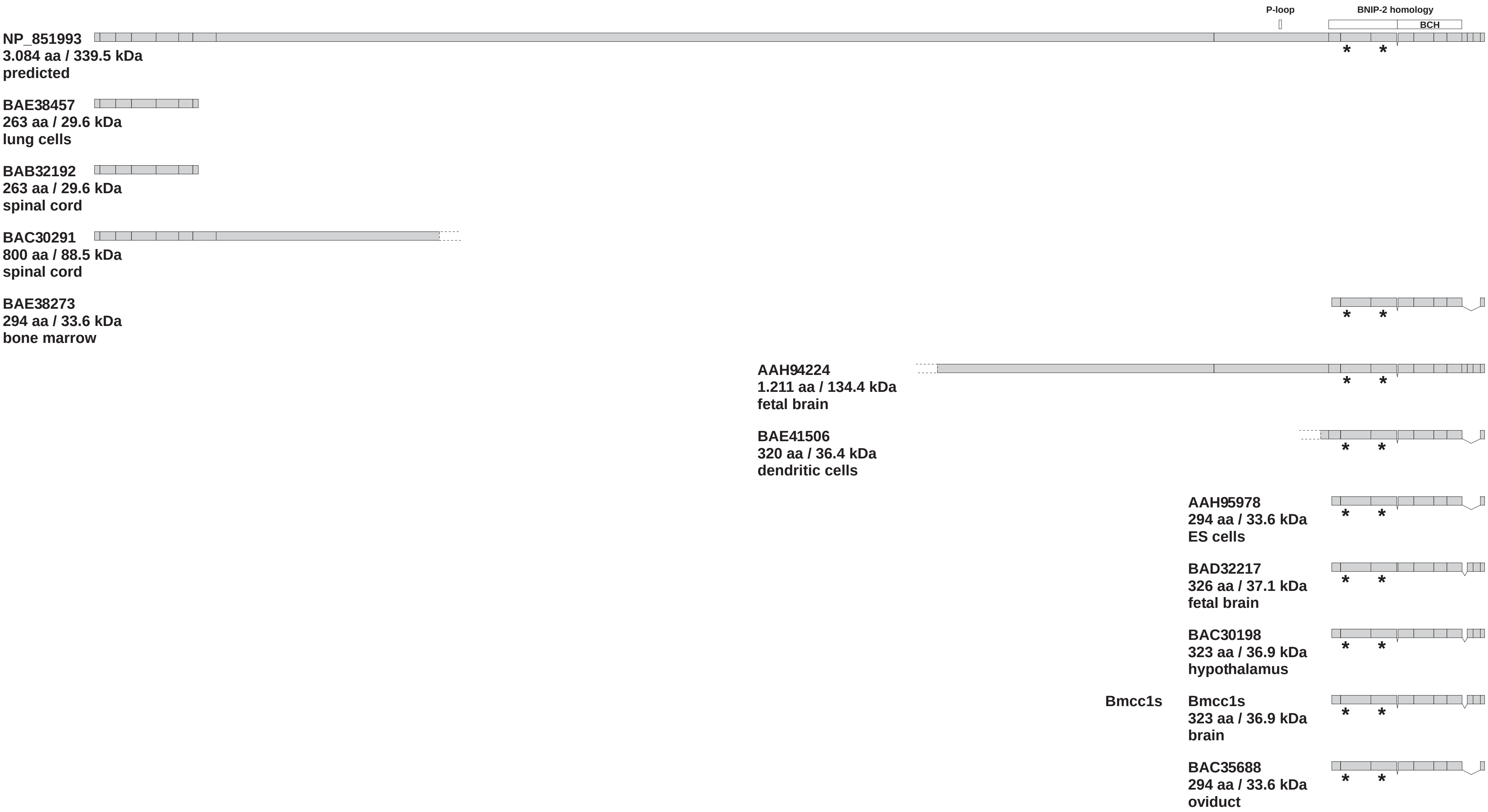

Supplement: Figure S1 — Mouse Bmcc1/Prune2 gene, transcripts and proteins. (A) Schematic representation of mouse Bmcc1 gene. All exons and introns are at scale, unless indicated. Insert at exons 7a/7b indicates the orthologous exon 4 of the human PCA3 gene on the opposite strand, which overlaps with exon 7a. A & B. Exons are boxed, in black for the coding sequence and in white for the 5′ and 3′ non-coding sequences. Alternative start and stop codons are indicated. (B) Schematic representation of mouse Bmcc1 transcripts. Scale is as in A, and transcripts are given with their accession number, size, library type, and exon composition. Solid bar under exon 21 indicates DNA arrays probe set. Primers for 5′ RACE experiments are indicated by arrows under exons 11/12 and 21. Dashes indicate reading frames that are still open. (C) Schematic representation of mouse protein Bmcc1 protein isoforms encoded by the corresponding transcripts shown in B. Proteins are at scale, with their accession number, size, and library type. Corresponding coding exons are boxed in light gray. Dashes indicate that protein may be longer. Conserved domains described in [36] are indicated on the top of the longest protein, as the antigenic peptides (asterisks) used to generate the Bmcc1s antiserum. In comparison to the human sequences presented in figure S2, Bmcc1 displays an additional exon 7a, generating specific C-termini in the N-ter proteins. Second, translation of mouse C-ter Bmcc1 proteins is initiated at the ATG initiation codon within exon 10, while in human BMCC1 it starts either in exon 9b or in exon 9c. Consequently, all Bmcc1 C-ter proteins share the same N-terminus, which differs in human. Finally, exon 7a overlaps the orthologous human PCA3 exon 4 on the opposite strand, while human PCA3 coding-exons located in intron 6 do not overlap with BMCC1 exons [36]. (PDF) [file pone.0035488.s001.pdf]
