# Supplementary figures and images for: Bmcc1s, a Novel Brain-Isoform of Bmcc1, Affects Cell Morphology by Regulating MAP6/STOP Functions
Source: PLoS One. 2012 Apr 16;7(4):e35488. doi: 10.1371/journal.pone.0035488 (PMC3327665; doi:10.1371/journal.pone.0035488)

## A) Human BMCC1 / PRUNE2 gene (295 kbp)

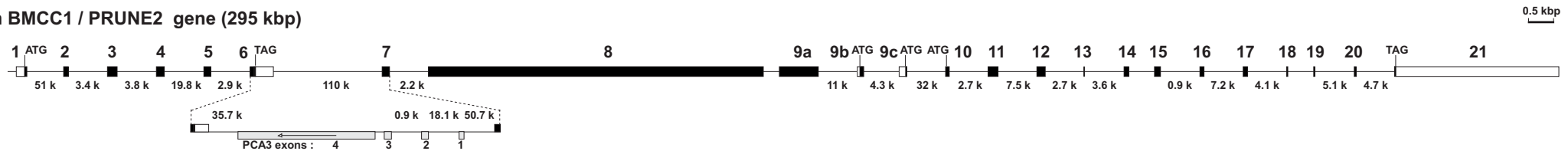

## B) Human Bmcc1 / Prune2 cDNAs

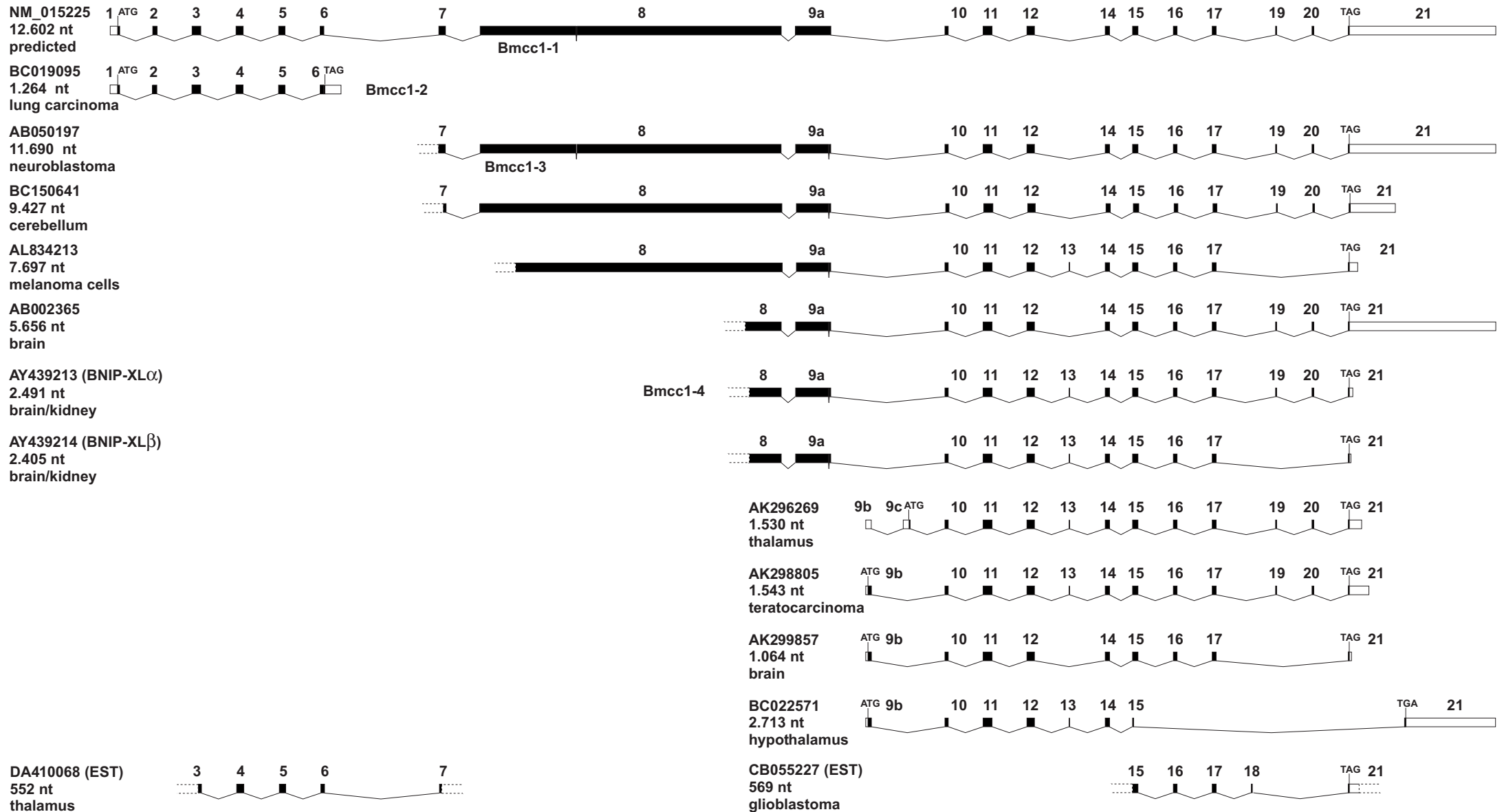

C) Human Bmcc1 / Prune2 proteins

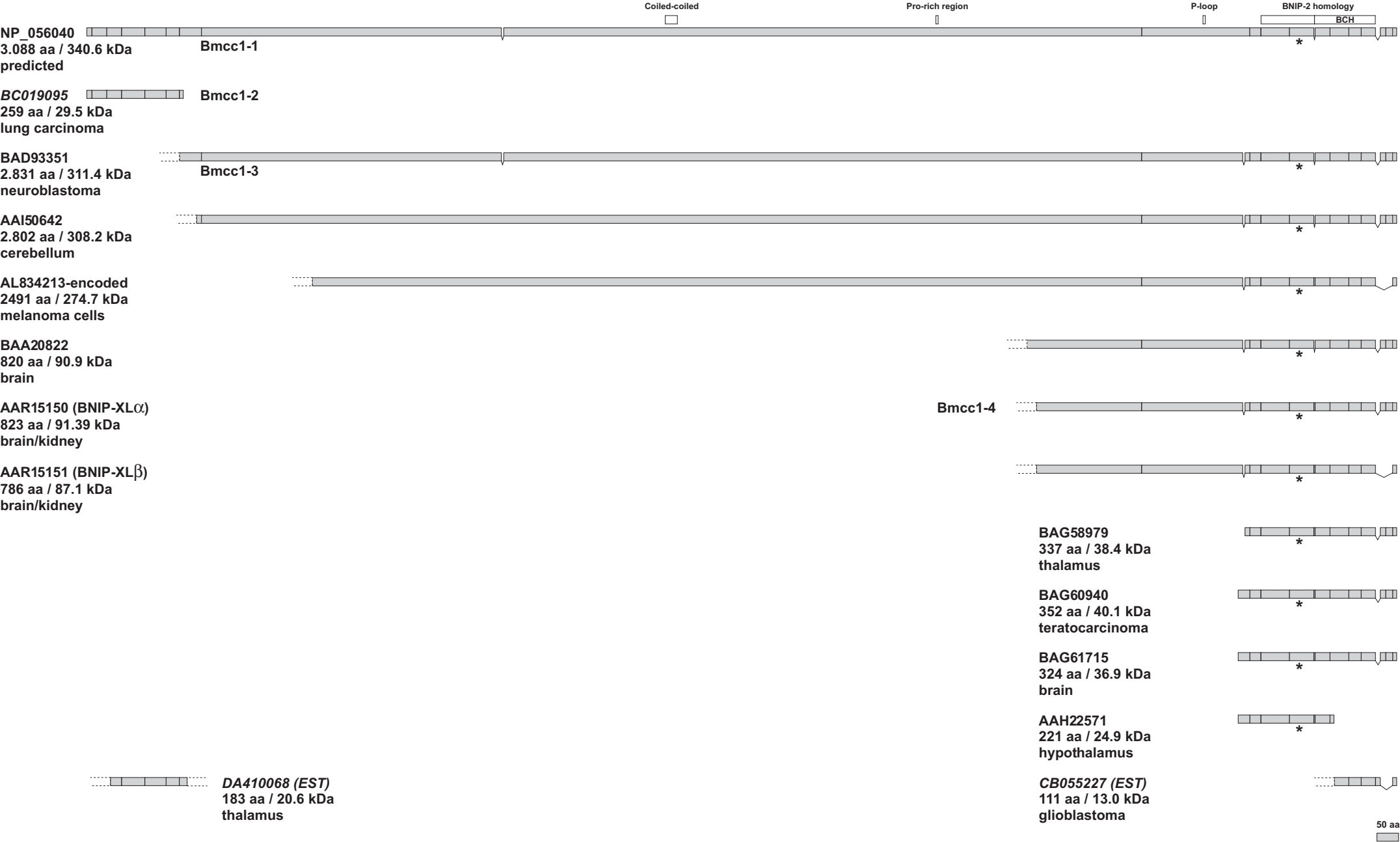

Supplement: Figure S2 — Human BMCC1/PRUNE2 gene, transcripts and proteins. (A) Schematic representation of the BMCC1 gene. All indicated exons and introns are at scale. Insert at intron 6 indicates the four PCA3 gene exons on the opposite strand. (A,B) Exons are boxed in black for the coding sequence and in white for the 5′ and 3′ non-coding sequences. Alternative start and stop codons are indicated. B. Schematic representation of human BMCC1 transcripts. Scale is as in A, and transcripts are given with their accession number, size, library type, and exon composition. Dashes indicate still opened reading frames. C. Schematic representation of human BMCC1 protein isoforms encoded by the corresponding transcripts shown in B. Proteins are at scale, with their accession number, size, and library type. Corresponding coding exons are boxed in light gray. Dashes indicate that protein may be longer. Bmcc1-1 to Bmcc1-4 are described in [36]. Accession numbers of the partial transcripts (EST) linking exons 1–6 to the remaining exons, or demonstrating the presence of the ortholog of mouse exon 18 in human transcripts and gene are in italics. Conserved domains described in [36] are indicated at the top of the longest protein, as well as the conserved epitope (asterisk) used to generate Bmcc1 antiserum. (PDF) [file pone.0035488.s002.pdf]

**Brain**

**Kidney**

**Liver**

**Heart**

**Thymus**

**Lung**

**Bmcc1s**

**Hprt**

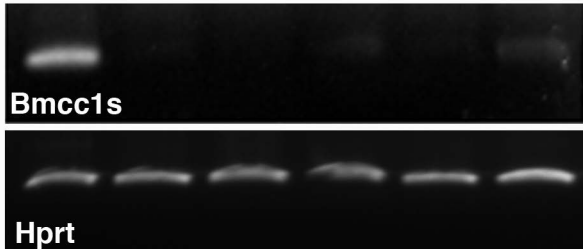

Supplement: Figure S3 — Expression profile of Bmcc1s. RT-PCR using total RNA extracted from various mouse tissues on the 3′ end of the Bmcc1s 3′UTR. Amplification occurred mainly in the brain, demonstrating that Bmcc1s expression is highly specific to this organ. Hprt amplification was used as an internal control. (PDF) [file pone.0035488.s003.pdf]

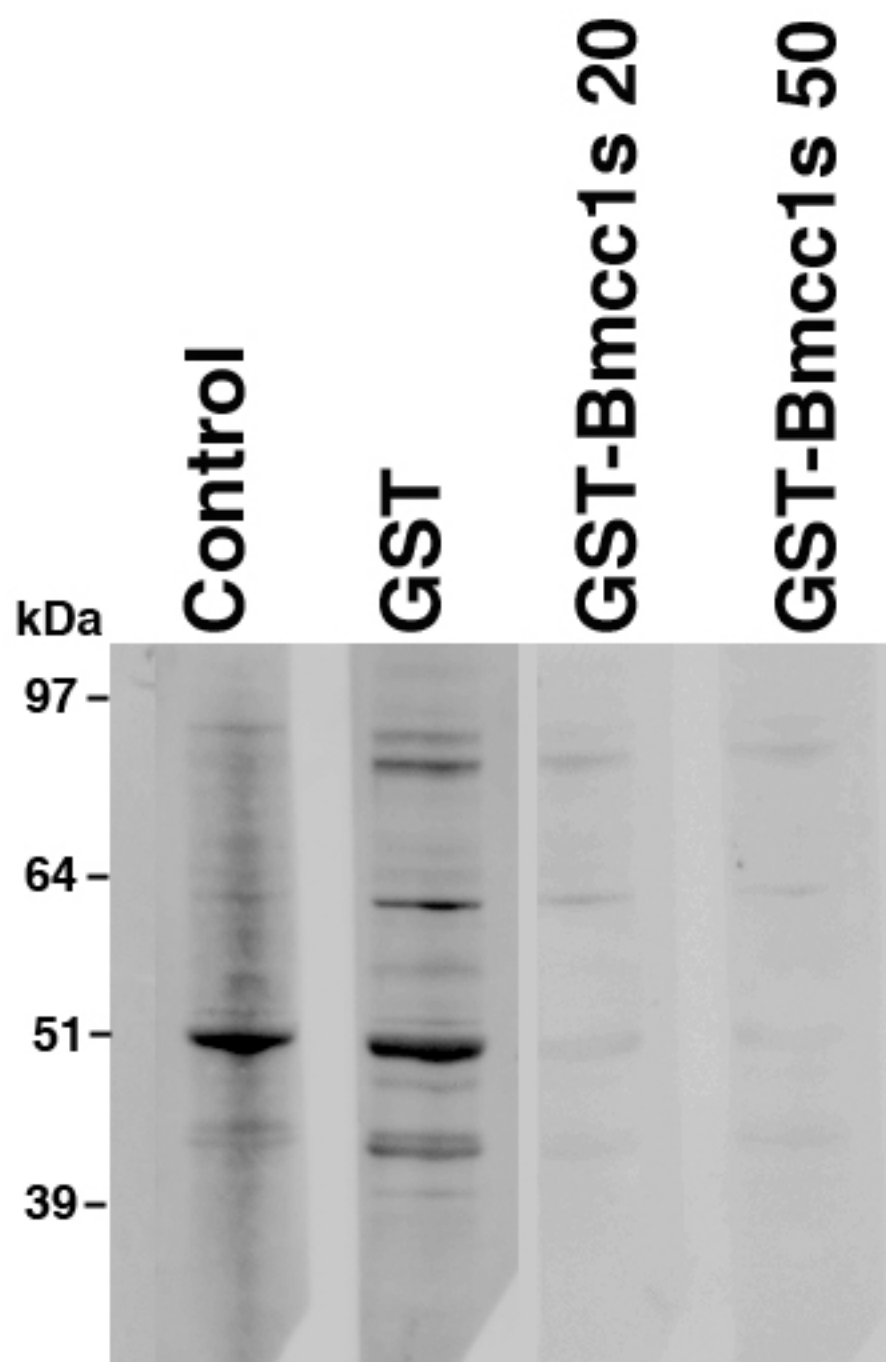

**Bmcc1s**

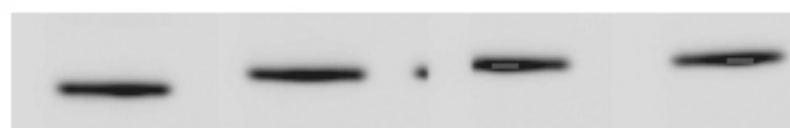

**GAPDH**

Supplement: Figure S4 — Specificity test of the Bmcc1s antiserum. Immunoblotting of adult mouse cortex proteins with the Bmcc1s antiserum (Control), or the antiserum preincubated on sepahrose bound GST or increasing concentrations of sepharose bound GST-Bmcc1s. GAPDH expression is shown as a loading reference. (PDF) [file pone.0035488.s004.pdf]

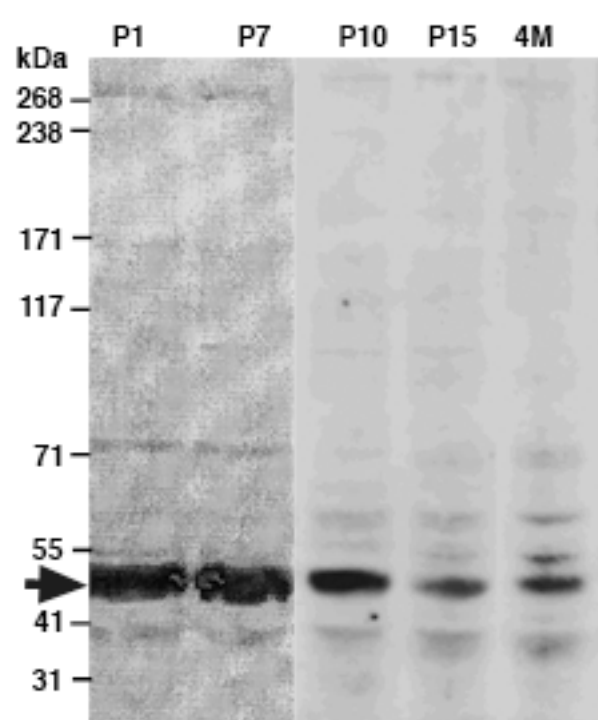

Supplement: Figure S5 — Immunodetection of Bmcc1s in the post-natal developing brain. Immunoblot of endogenous Bmcc1 isoforms in mouse brain lysates of post-natal day (P) 1 to 4 months, using Bmcc1 antiserum. A major 50 kDa band (arrow) corresponding to Bmcc1s was detected at all stages. (PDF) [file pone.0035488.s005.pdf]

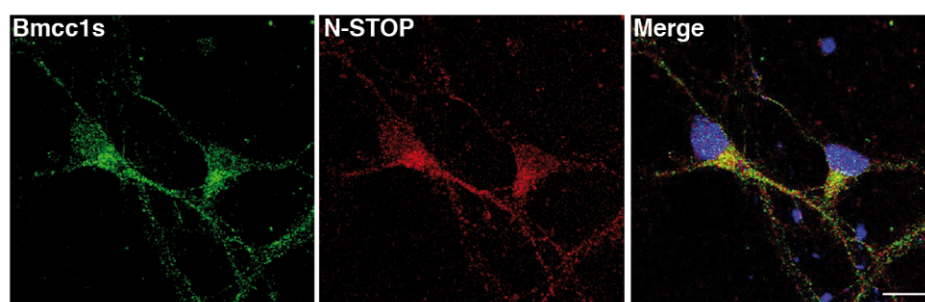

Supplement: Figure S6 — Bmcc1s colocalizes with the neuronal MAP6 isoform N-STOP in primary neurons. Confocal section images of primary neurons immunostained for Bmcc1s (green) and N-STOP (red) using the monoclonal antibody 175 [29]. Bar: 10 µm. (PDF) [file pone.0035488.s006.pdf]
